# Supplementary material for: Duplication events downstream of IRX1 cause North Carolina macular dystrophy at the MCDR3 locus
Source: Sci Rep. 2017 Aug 8;7:7512. doi: 10.1038/s41598-017-06387-6 (PMC5548758; doi:10.1038/s41598-017-06387-6)
Supplement: Supplementary file 1 — Supplementary Information [file 41598_2017_6387_MOESM1_ESM.pdf]

SUPPLEMENTARY MATERIALS

Duplication events downstream of *IRX1* cause North Carolina macular dystrophy at the MCDR3 locus

Valentina Cipriani,[1,2,3,§] Raquel S Silva,[1,2,§] Gavin Arno,[1,2] Nikolas Pontikos,[1,3] Ambreen Kalhoro,[1,2] Sandra Valeina,[4] Inna Inashkina,[5] Mareta Audere,[5,6] Katrina Rutka,[5,6] Bernard Puech,[7] Michel Michaelides,[1,2] Veronica van Heyningen,[1] Baiba Lace,[5,8] Andrew R Webster,[1,2] Anthony T Moore[1,2,9]

[1]UCL Institute of Ophthalmology, London, UK, [2]Moorfields Eye Hospital, London, UK, [3]UCL Genetics Institute, London, UK, [4]Children's Clinical University Hospital, Riga, Latvia, [5]Latvian Biomedical Research and Study Centre, Riga, Latvia, [6]Riga Stradins University, Riga, Latvia [7]Exploration de la Vision et Neuro-Ophtalmologie, Centre Hospitalier Universitaire, Lille, France [8]Centre Hospitalier de l'Université Laval, Québec, Canada, [9]Ophthalmology Department, UCSF School of Medicine, San Francisco, CA, USA

[§]These authors contributed equally to this work

Supplementary Figures S1-S11

Supplementary Tables S1-S2

Supplementary Figure S1 Pedigree structures of families 1-13. Black solid and open symbols indicate affected and unaffected individuals respectively. Grey solid symbols indicate family members with unknown disease status. SNP, aCGH and WGS indicate the individual was studied using Illumina single-nucleotide polymorphism array data, array-based comparative genomic hybridization and whole-genome sequencing, respectively. The symbols +/- and -/- indicate presence and absence of either the 43 kb or 45 kb duplications at the 5p15 locus, or the previously reported single nucleotide V2 variant upstream of *PRDM13* at the 6q16 locus.[1]

Family 1 (GC19806)

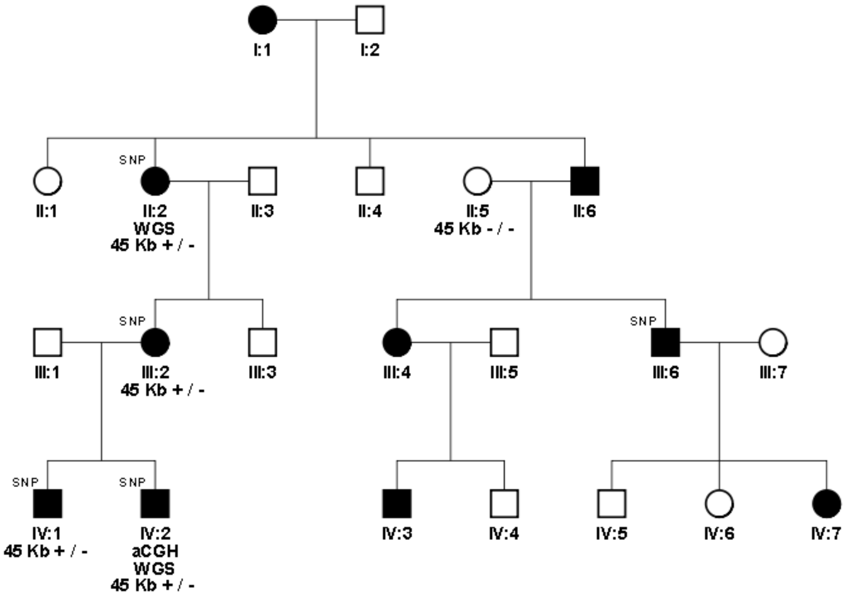

Family 2 (GC15626)

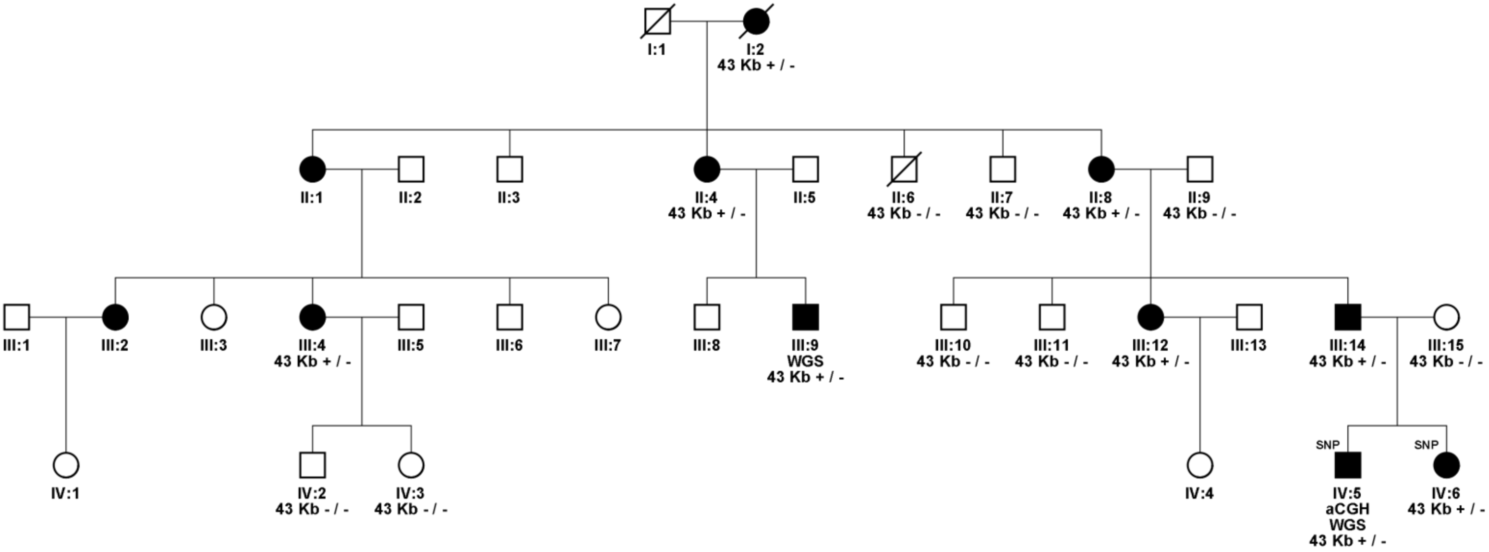

Family 3 (GC15119)

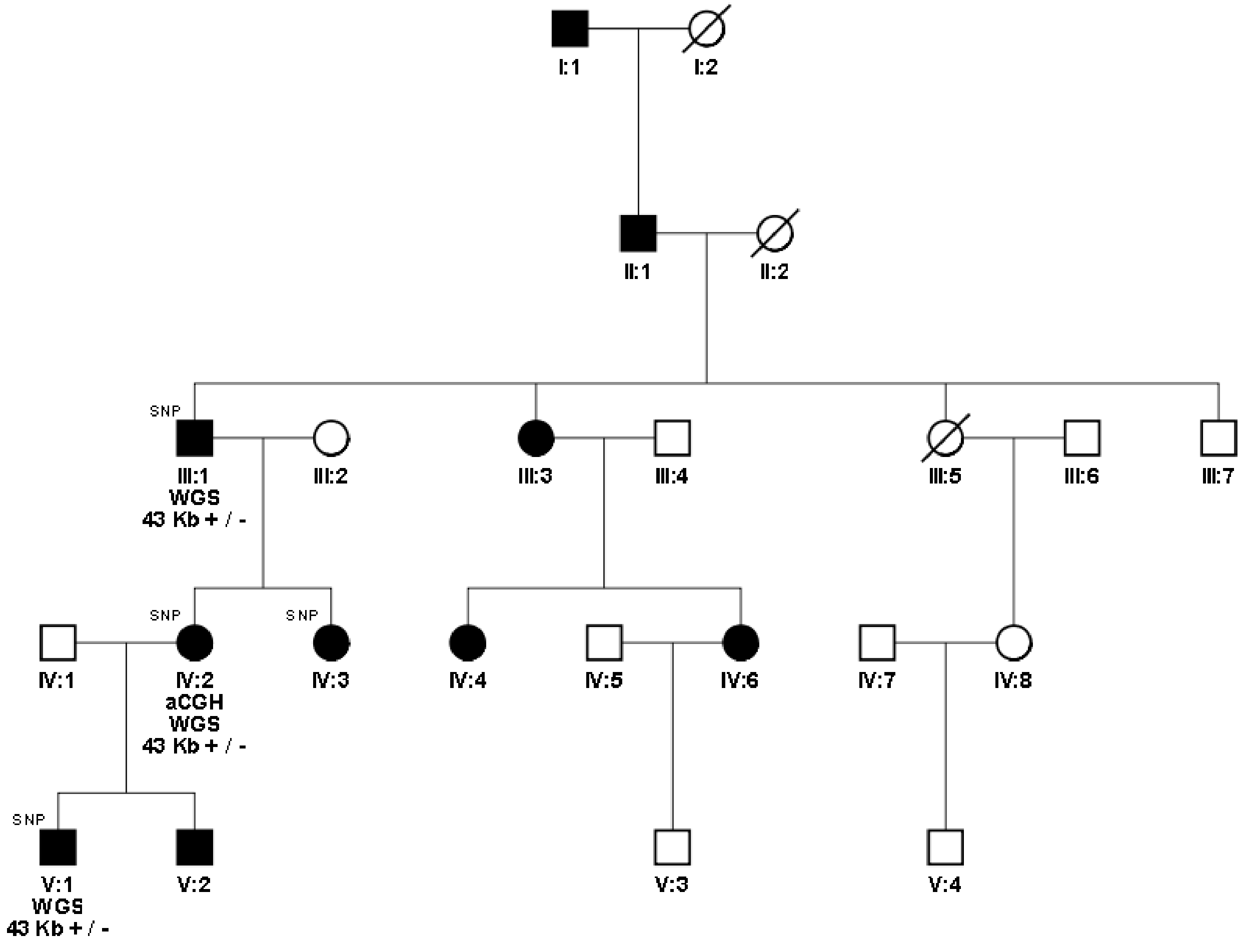

Family 4 (GC13840)

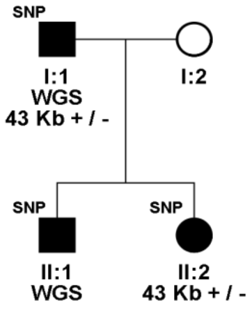

Family 5 (GC19075)

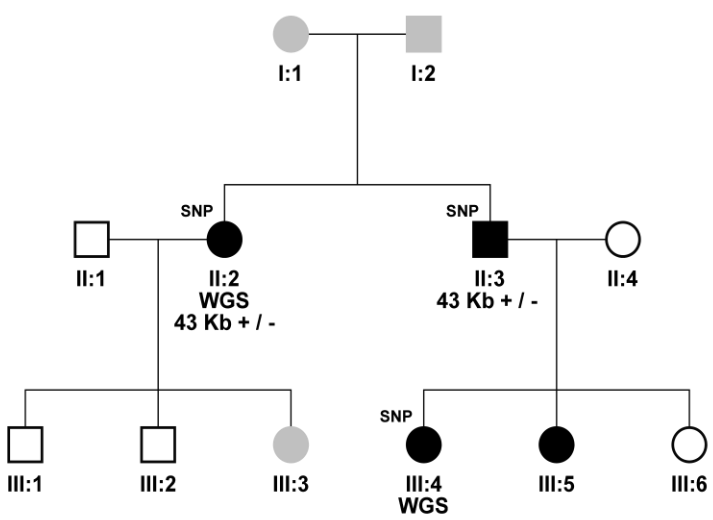

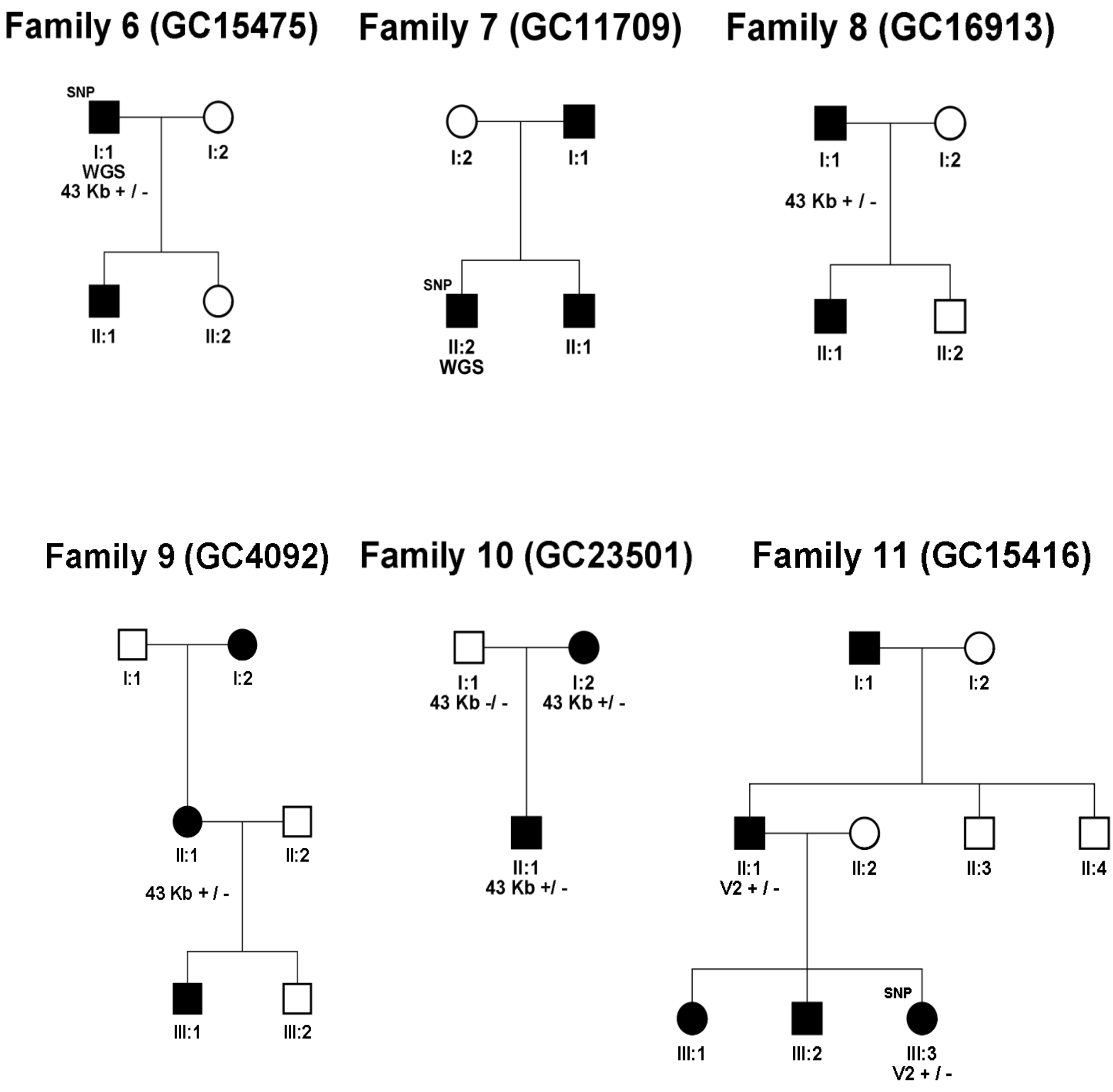
Family 6 (GC15475)
SNP
I:1
WGS
43 Kb + / -
I:2
II:1
II:2
Family 7 (GC11709)
I:2
I:1
SNP
II:2
WGS
II:1
Family 8 (GC16913)
I:1
I:2
43 Kb + / -
II:1
II:2
Family 9 (GC4092)
I:1
I:2
II:1
II:2
43 Kb + / -
III:1
III:2
Family 10 (GC23501)
I:1
43 Kb -/ -
I:2
43 Kb +/ -
II:1
43 Kb +/ -
Family 11 (GC15416)
I:1
I:2
II:1
V2 + / -
II:2
II:3
II:4
III:1
III:2
SNP
III:3
V2 + / -

Family 12 (GC3722)

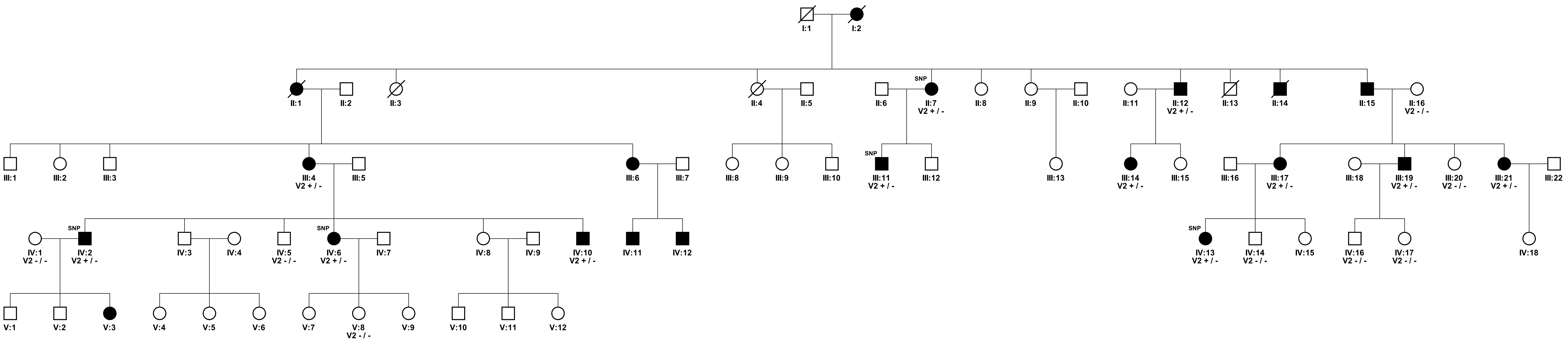

Family 13 (GC17225)

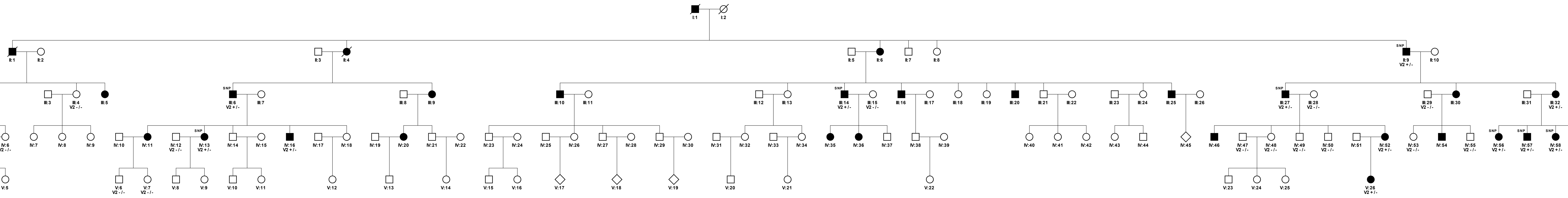

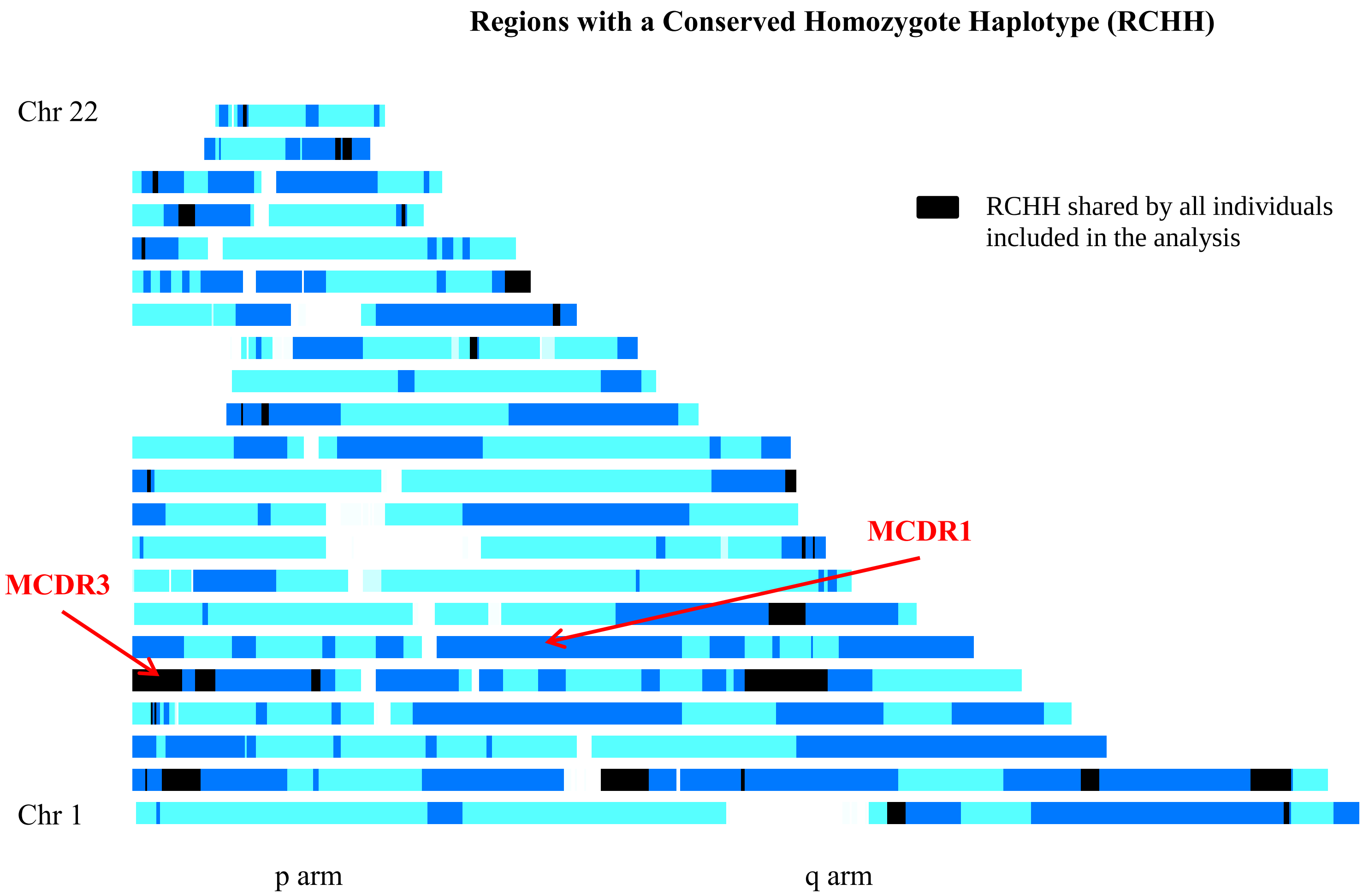

Supplementary Figure S2 Identification of the disease candidate regions for family 1 (GC19806) using the Homozygosity Haplotype (HH) approach.[2] Five affected family members were included in the analysis. A densitogram of the genomic Regions with a Conserved Homozygosity Haplotype (RCHH) is depicted. The darker the colour, the more individuals share a HH in the region. Black regions indicate RCHH that are shared by all 5 affected family members included in the analysis.

Regions with a Conserved Homozygote Haplotype (RCHH)

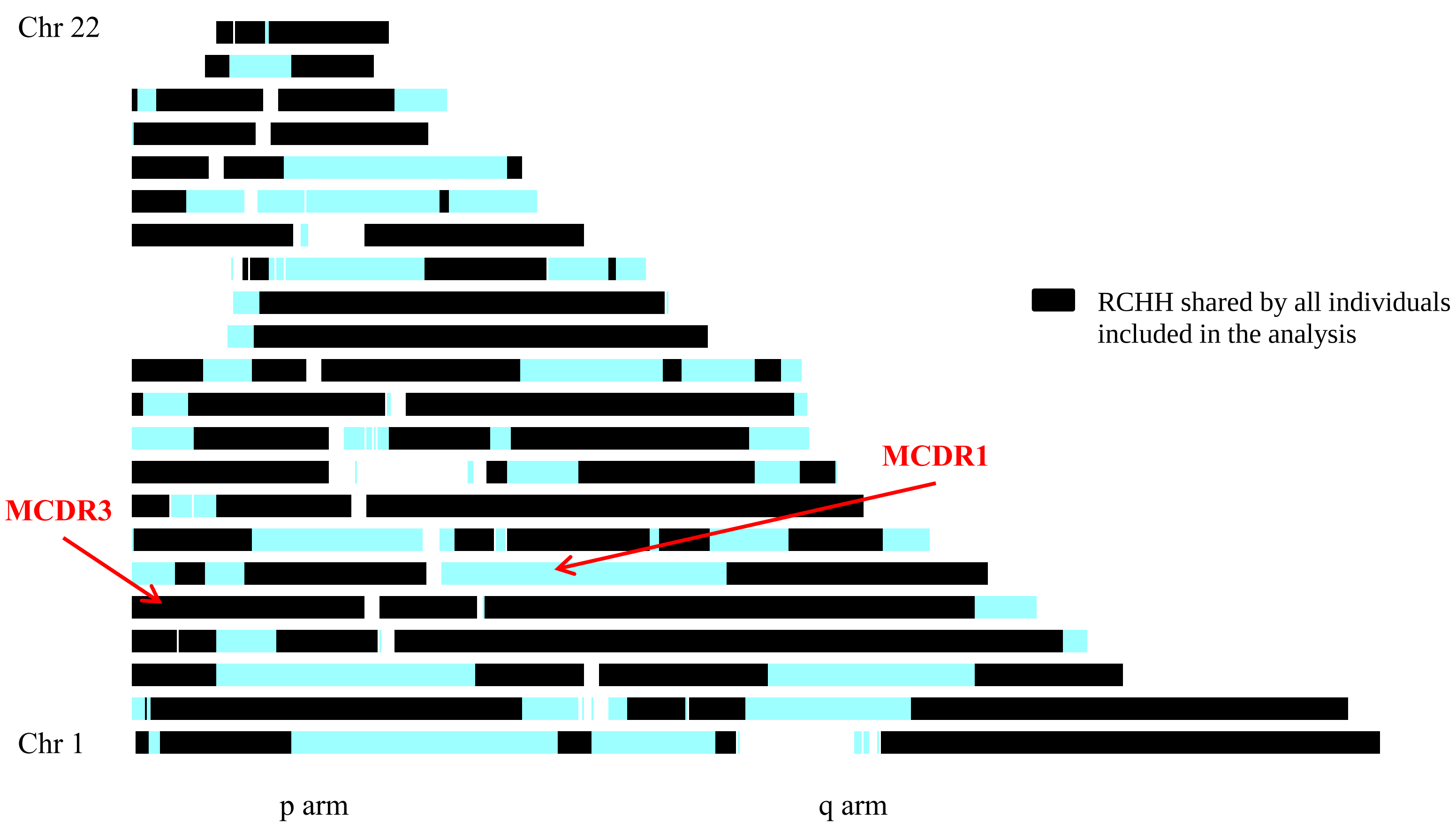

Supplementary Figure S3 Identification of the disease candidate regions for family 2 (GC15626) using the Homozygosity Haplotype (HH) approach.[2] Two affected family members were included in the analysis. A densitogram of the genomic Regions with a Conserved Homozygosity Haplotype (RCHH) is depicted. The darker the colour, the more individuals share a HH in the region. Black regions indicate RCHH that are shared by both affected family members included in the analysis.

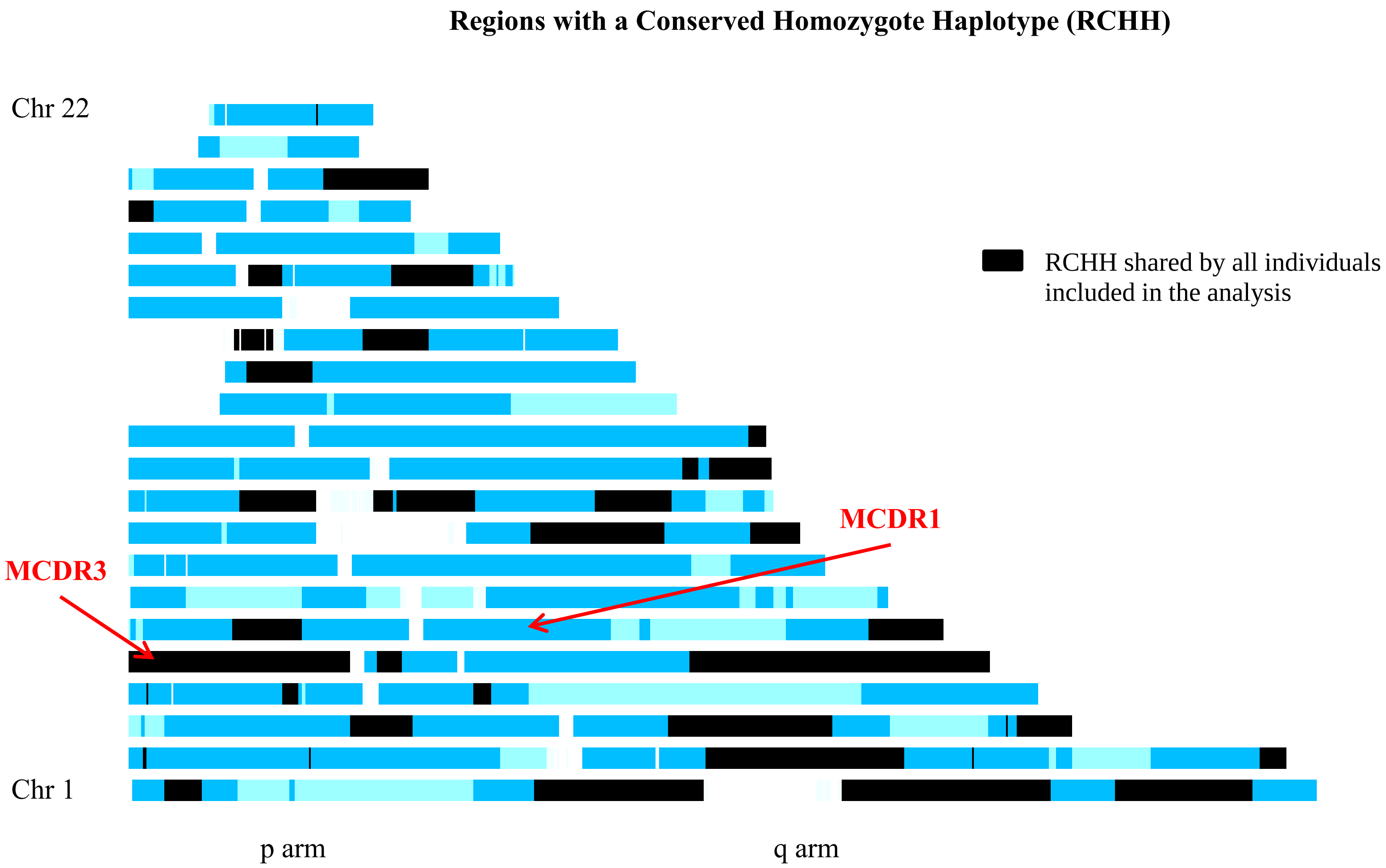

Supplementary Figure S4 Identification of the disease candidate regions for family 3 (GC15119) using the Homozygosity Haplotype (HH) approach.[2] Four affected family members were included in the analysis. A densitogram of the genomic Regions with a Conserved Homozygosity Haplotype (RCHH) is depicted. The darker the colour, the more individuals share a HH in the region. Black regions indicate RCHH that are shared by all 4 affected family members included in the analysis.

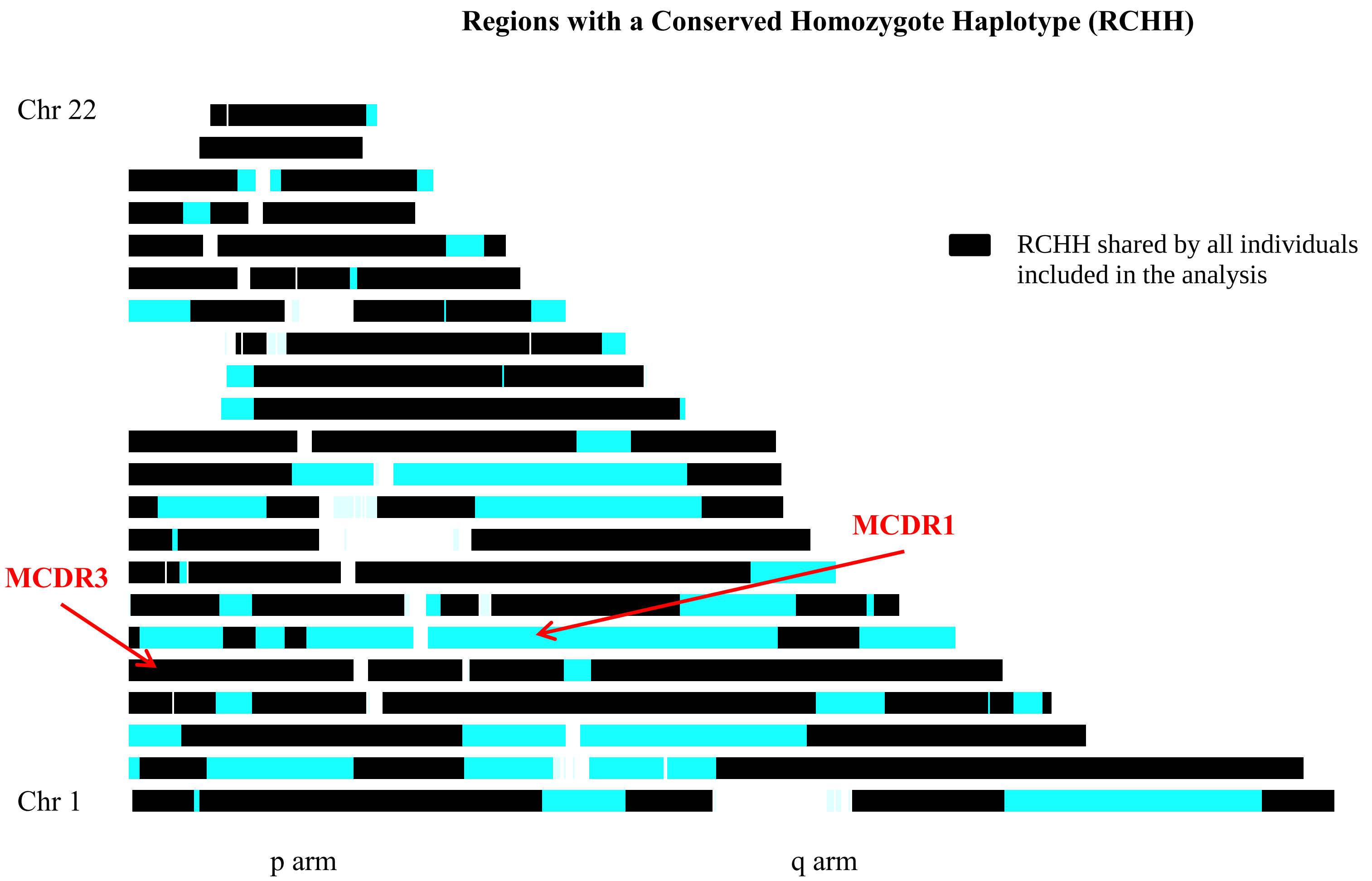

Supplementary Figure S5 Identification of the disease candidate regions for family 4 (GC13840) using the Homozygosity Haplotype (HH) approach.[2] Three affected family members were included in the analysis. A densitogram of the genomic Regions with a Conserved Homozygosity Haplotype (RCHH) is depicted. The darker the colour, the more individuals share a HH in the region. Black regions indicate RCHH that are shared by all 3 affected family members included in the analysis.

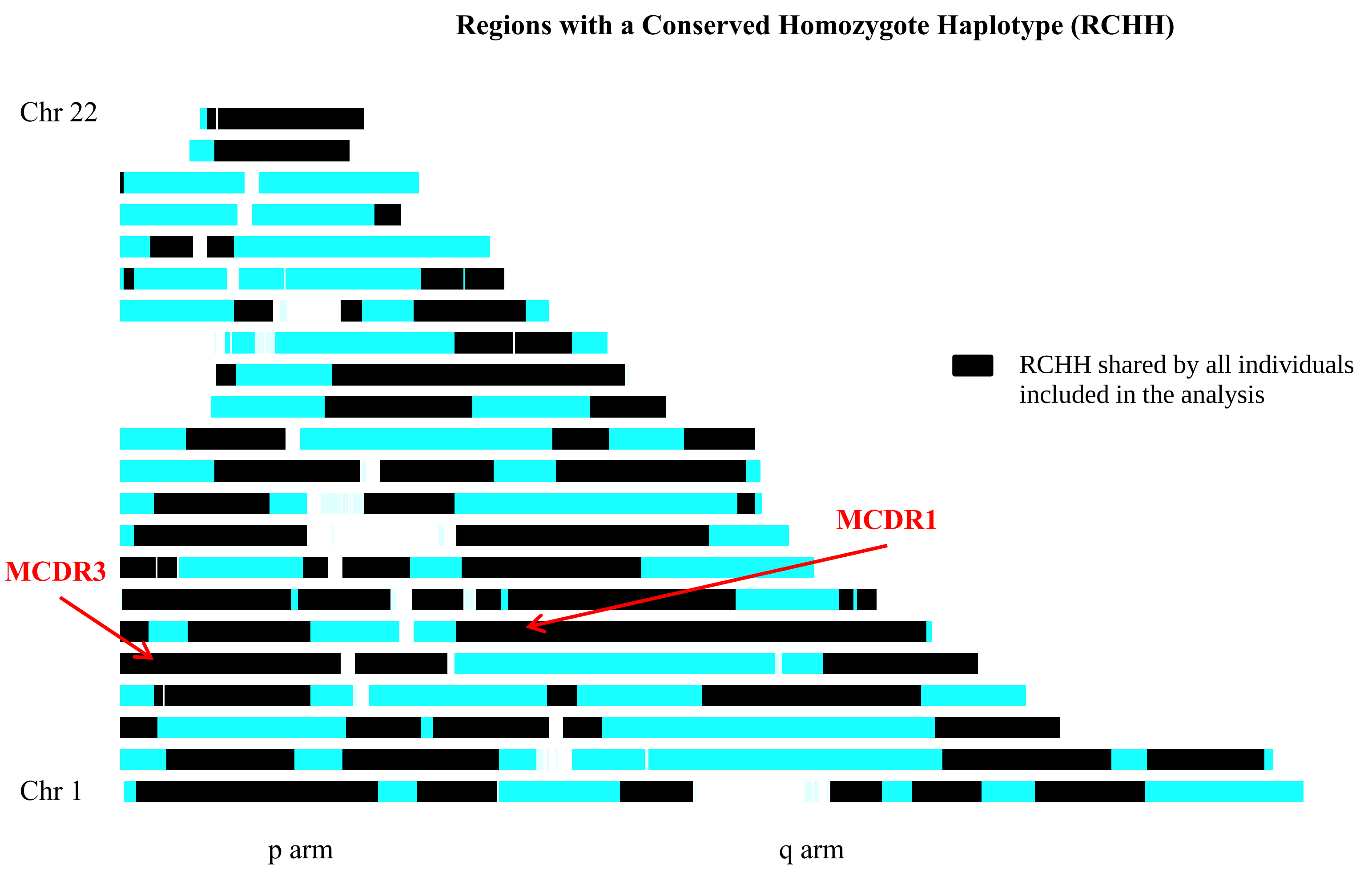

Supplementary Figure S6 Identification of the disease candidate regions for family 5 (GC19075) using the Homozygosity Haplotype (HH) approach.[2] Three affected family members were included in the analysis. A densitogram of the genomic Regions with a Conserved Homozygosity Haplotype (RCHH) is depicted. The darker the colour, the more individuals share a HH in the region. Black regions indicate RCHH that are shared by all 3 affected family members included in the analysis.

Regions with a Conserved Homozygote Haplotype (RCHH)

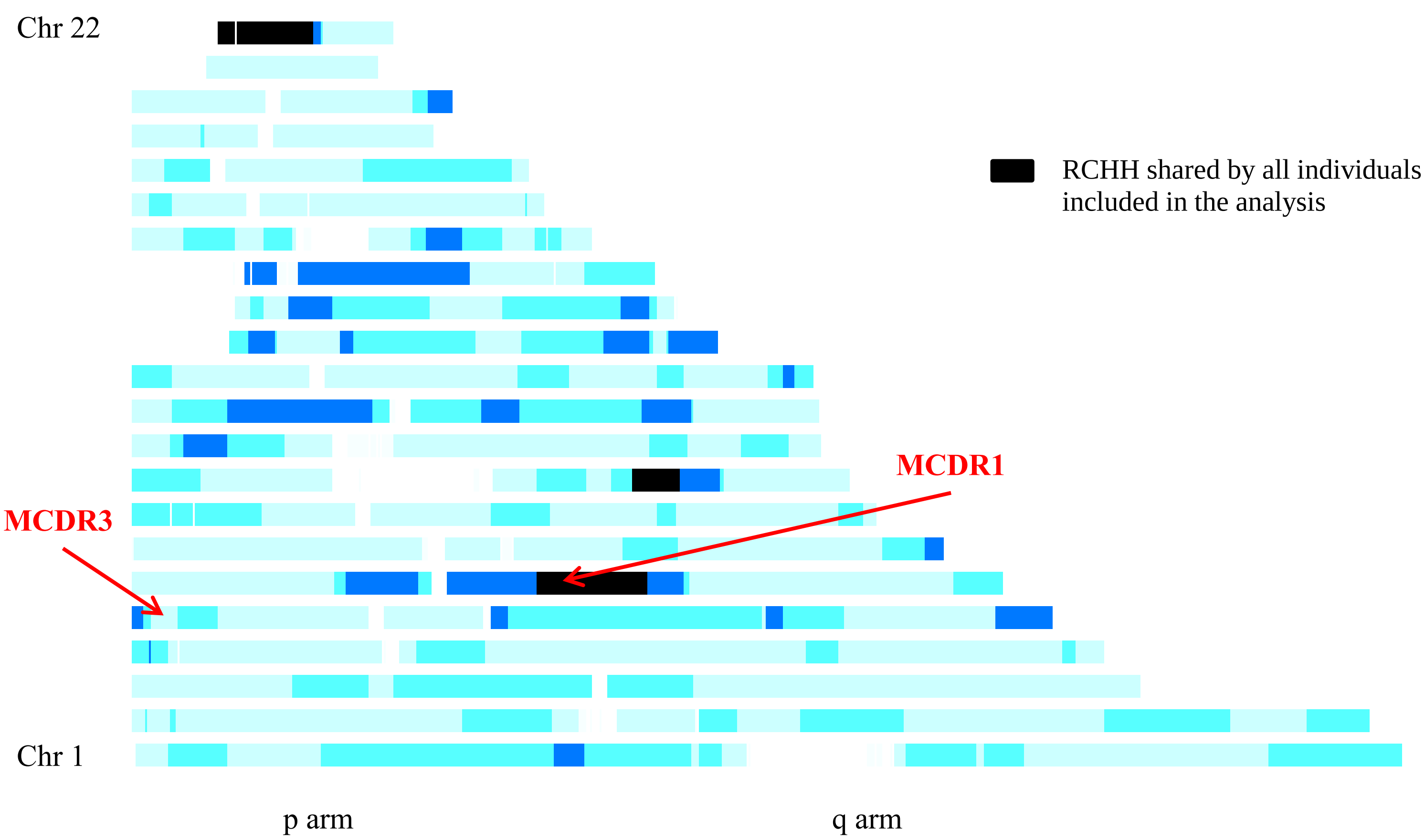

Supplementary Figure S7 Identification of the disease candidate regions for family 12 (GC3722) using the Homozygosity Haplotype (HH) approach.[2] Five affected family members were included in the analysis. A densitogram of the genomic Regions with a Conserved Homozygosity Haplotype (RCHH) is depicted. The darker the colour, the more individuals share a HH in the region. Black regions indicate RCHH that are shared by all 5 affected family members included in the analysis.

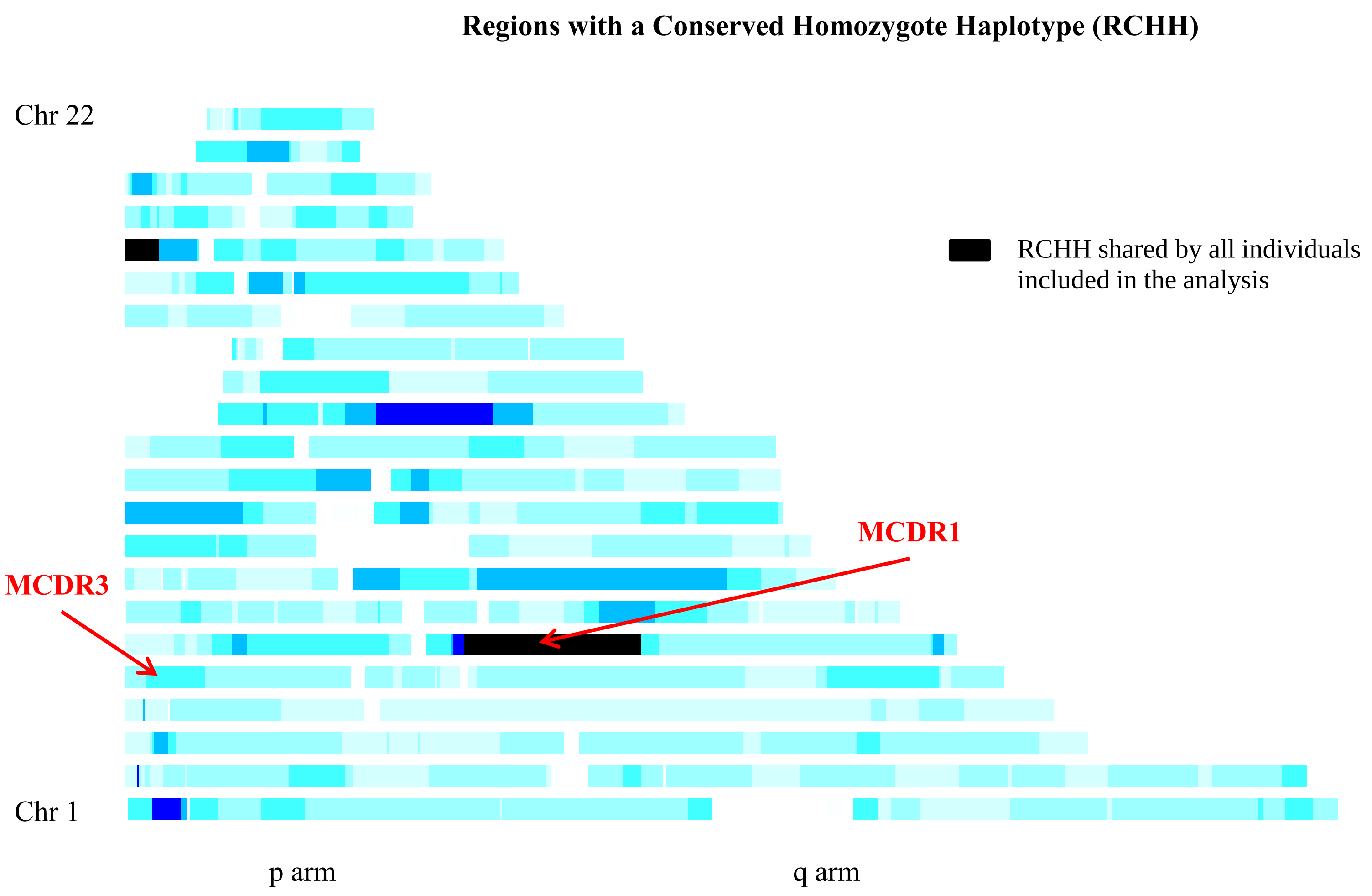

Supplementary Figure S8 Identification of the disease candidate regions for family 13 (GC17225) using the Homozygosity Haplotype (HH) approach.[2] Eight affected family members were included in the analysis. A densitogram of the genomic Regions with a Conserved Homozygosity Haplotype (RCHH) is depicted. The darker the colour, the more individuals share a HH in the region. Black regions indicate RCHH that are shared by all 8 affected family members included in the analysis.

Regions with a Conserved Homozygote Haplotype (RCHH)

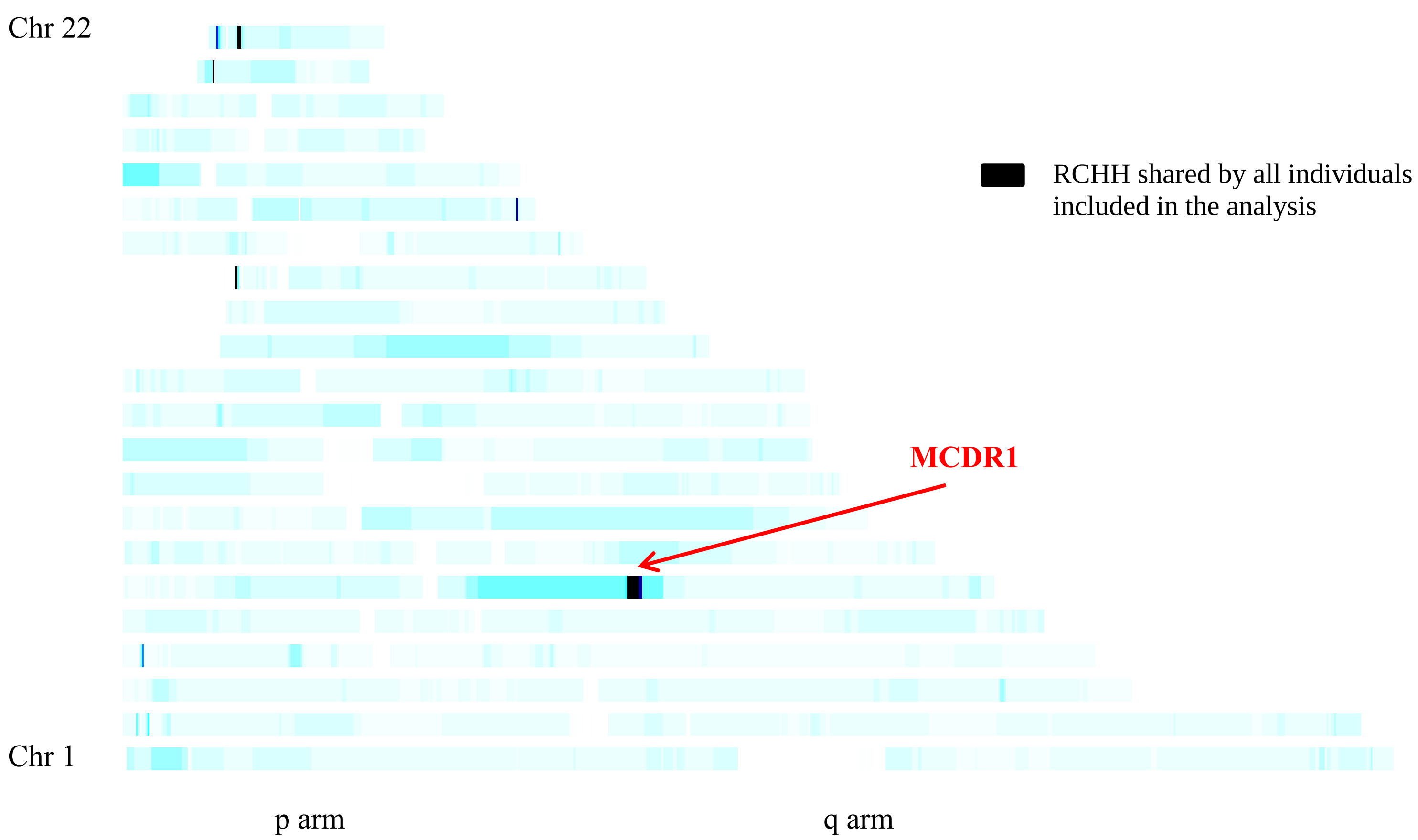

Supplementary Figure S9 Homozygosity Haplotype (HH) analysis[2] on a total of 14 affected individuals in 3 families (11-13) carrying the previously reported 6q16 V2 variant[1] and with available Illumina SNP array data. A densitogram of the genomic Regions with a Conserved Homozygosity Haplotype (RCHH) is depicted. The darker the colour, the more individuals share a HH in the region. All 14 genotyped individuals collectively shared a RCHH from GRCh37/hg19 coordinate 98962591 (rs150396) to 101468591 (rs1321204) at the MCDR1 locus.

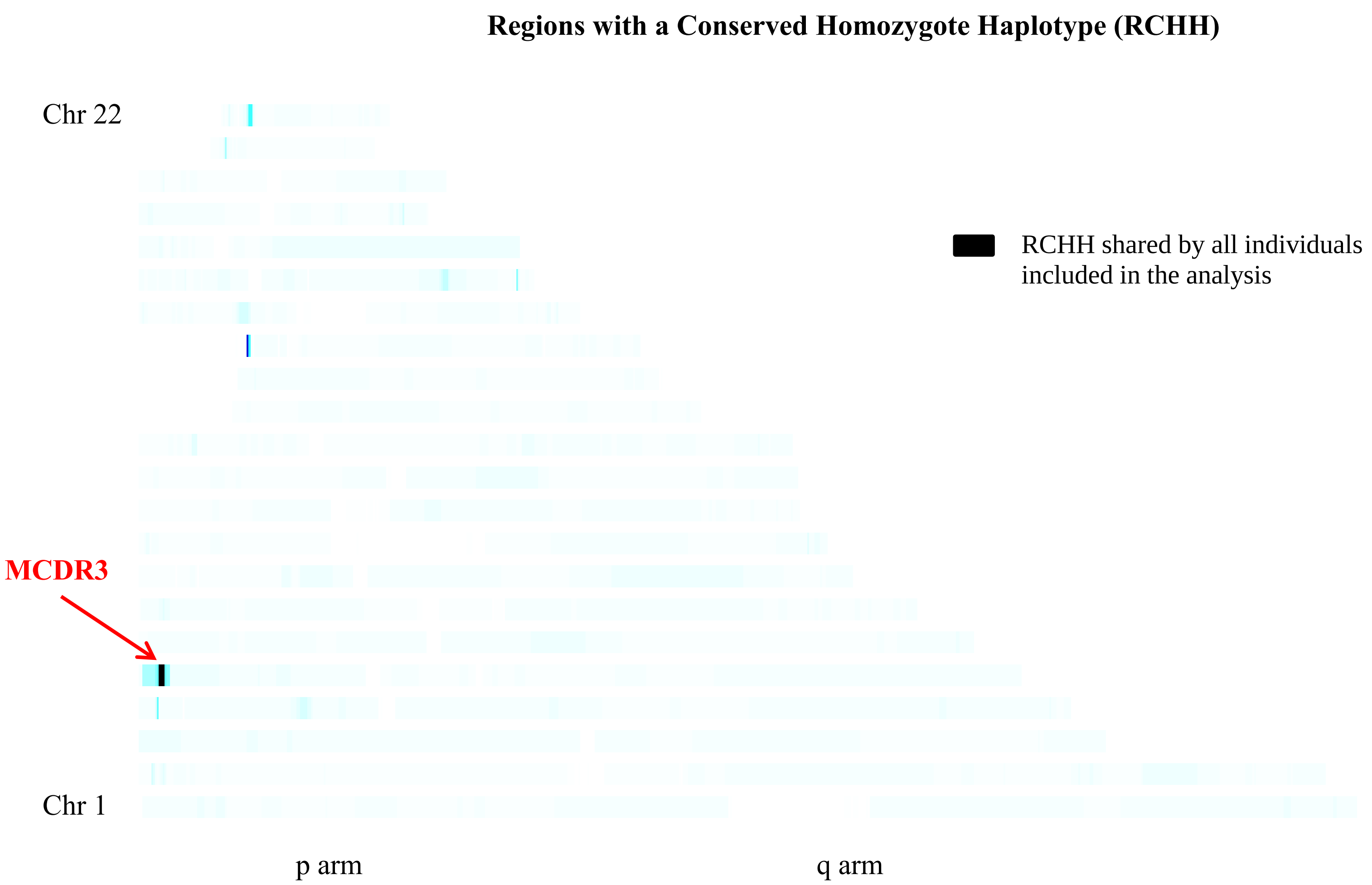

Supplementary Figure S10 Homozygosity Haplotype (HH) analysis[2] on a total of 14 affected individuals in 6 families (2-7) carrying the 5p15 43 kb duplication reported in this study and with available Illumina SNP array data. A densitogram of the genomic Regions with a Conserved Homozygosity Haplotype (RCHH) is depicted. The darker the colour, the more individuals share a HH in the region. All 14 genotyped individuals collectively shared a RCHH from GRCh37/hg19 coordinate 4325295 (rs155353) to 5210315 (rs7704912) at the MCDR3 locus.

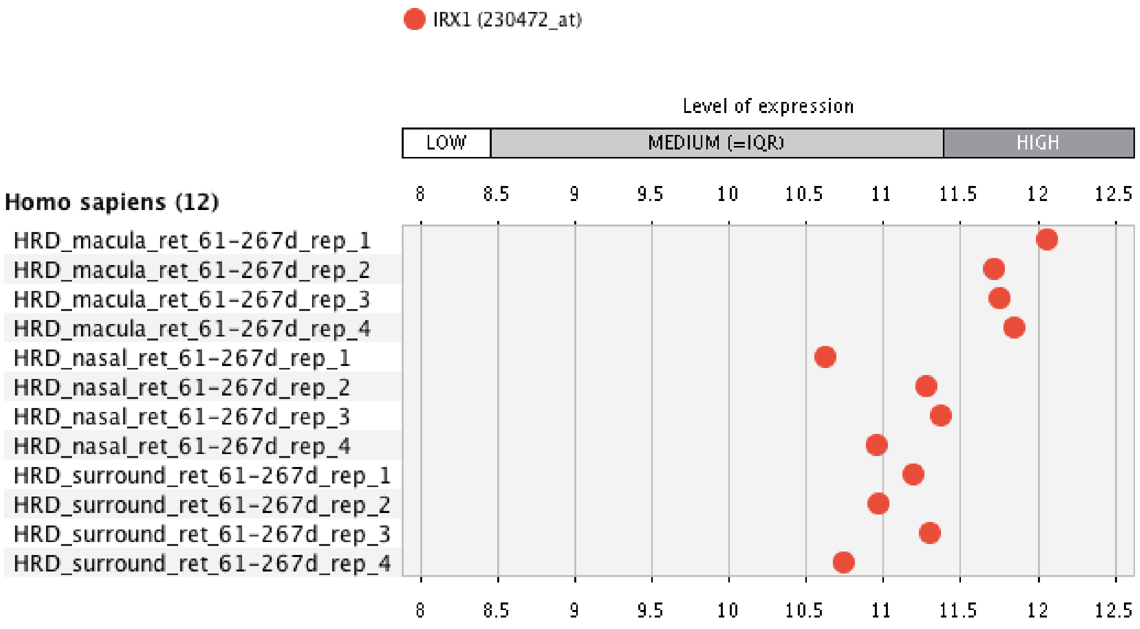

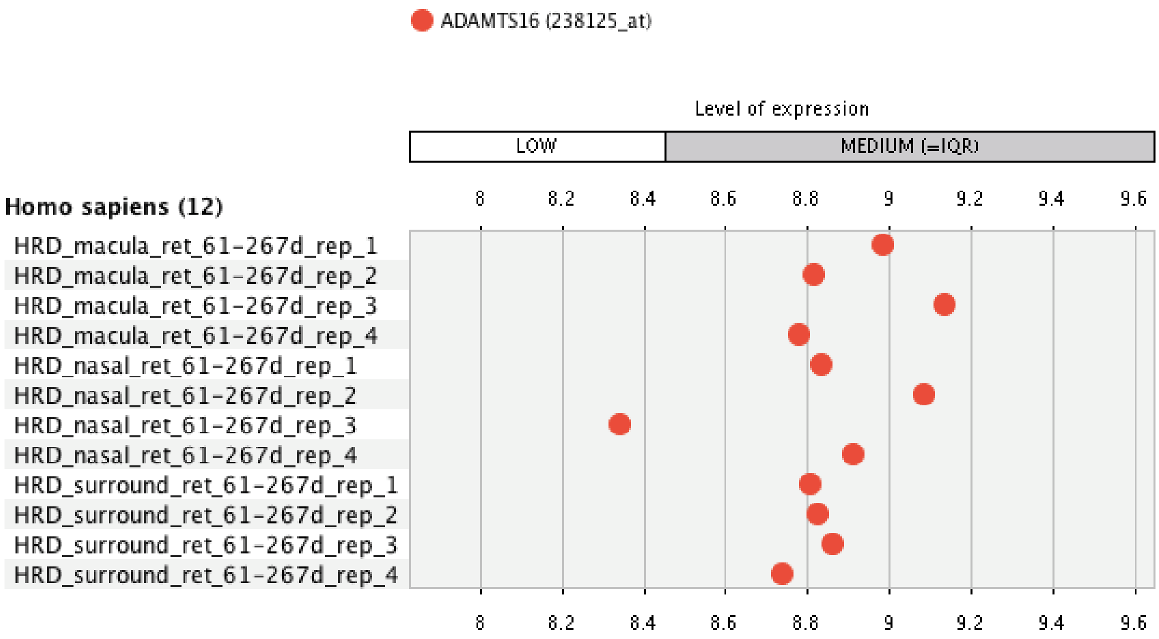

Supplementary Figure S11 Human fetal expression of *IRX1* and *ADAMTS16* in macula, nasal and surrounding retina. GENEVESTIGATOR[3] was used to query microarray datasets for expression of genes surrounding the duplication events at the MCDR3 locus. Fetal tissue used was 19-20 weeks post gestation.[4] *IRX1* is highly expressed in fetal macula, whereas *ADAMTS16* is moderately expressed without geographic specificity.

Supplementary Table S1 Region with a Conserved Homozygosity Haplotype[1] (HH) at the MCDR1 locus shared by 14 affected individuals from families 11-13 with available Illumina SNP array data. The HH is a type of haplotype described by the homozygous SNPs only (all heterozygous SNPs are removed) and, therefore, can be uniquely determined on each chromosome. Genotypes are displayed vertically per each individual (A=AA, B=BB, blank cells=AB). The start and end of the shared HH are marked with a red bold line.

Chromosome	Physical position (hg19)	Genetic map	rs number	Allele A frequency	Family 11 (GC15416)	Family 12 (GC3722)					Family 13 (GC17225)							
					III:3	III:11	IV:2	II:7	IV:6	IV:13	IV:57	IV:58	II:9	III:27	IV:56	III:6	IV:13	III:14
6	98935791	103.6579	rs1494779	0.222		B	B	B	B	B						A	A	
6	98936578	103.6583	rs9321067	0.071	B	B	B		B	B	B	B	B	B	B	B	B	B
6	98938023	103.767	rs17058578	0.131	B	B	B	B	B	B	B	B	B	B	B	B	B	B
6	98938237	103.7861	rs9401901	0.477	B	B		B		B	B	B		B	B			
6	98938249	103.7872	rs9388508	0.165	B	B	B	B	B	B	B	B	B		B	B	B	B
6	98938834	103.8382	rs2227121	0.513	B	B		B			B	B	B	B	B			
6	98949550	103.845	rs158773	0.376	B	B		B			B	B	B		B	B		
6	98953633	103.8464	rs158774	0.65			A	A	A		B	B	B		B			
6	98959605	103.8494	rs158777	0.442				A			B	B	B	B	B		B	B
6	98962591	103.9024	rs150396	0.301				B			B	B	B	B	B	B	B	B
6	98971740	103.9045	rs1481449	0.031	B	B	B	B	B	B	B	B	B	B	B	B	B	B
6	98975810	103.9049	rs211215	0.695				A			A	A	A	A	A	A	A	A
6	98978496	103.9055	rs11961327	0.031		B	B	B	B	B	B	B	B	B	B	B	B	B
6	98980147	103.9057	rs211222	0.164													A	A
6	98991464	103.9078	rs17058667	0.299				B			B	B	B	B	B	B	B	B
6	98992369	103.9079	rs183316	0.292				B			B	B	B	B	B	B	B	B
6	99001499	103.9107	rs211227	0.889		A	A	A	A	A	A	A	A	A	A	A	A	A
6	99004191	103.9111	rs9372864	0.545	B	B	B		B	B							B	B
6	99013740	103.9183	rs2046174	0.832	B													
6	99017732	103.9544	rs17829357	0.115		B	B	B	B	B	B	B	B	B	B	B	B	B
6	99019431	103.9548	rs9491794	0.262						A							A	A
6	99032723	103.9592	rs1481440	0.788	A	A	A	A	A		A	A		A	A	A		
6	99033489	103.9596	rs11752460	0.823	B													
6	99034313	103.9611	rs1481438	0.622	B					B			B				B	B
6	99045749	103.9622	rs6929790	0.788	A	A	A	A	A		A	A		A	A	A		
6	99050825	103.9628	rs17830067	0.146	A													
6	99058170	103.9632	rs969540	0.138	A													
6	99076746	103.9642	rs17058737	0.847	B													
6	99092539	103.9699	rs17058761	0.156	A													
6	99124184	103.9844	rs11752997	0.854	A	A	A	A	A		A	A	A	A	A	A	A	A
6	99126082	103.9857	rs4472356	0.587	B					B			B			B		
6	99138875	103.9982	rs12204275	0.42	B	B	B	B	B		B	B		B	B		B	B
6	99139350	103.9989	rs6939572	0.593						B			B			B		
6	99140492	104.0015	rs6924761	0.159	B	B	B	B	B	B	B	B	B	B	B		B	B
6	99147074	104.0354	rs4424090	0.031	B	B	B	B	B	B	B	B	B	B	B	B	B	B
6	99170039	104.0781	rs9375604	0.934	A	A	A	A	A	A	A	A		A	A		A	A
6	99173425	104.0791	rs4529305	0.92	A	A	A	A	A	A				A			A	A
6	99175625	104.081	rs9388676	0.128	B	B	B	B	B	B	B	B	B		B	B	B	B
6	99195548	104.0849	rs11154467	0.717		A	A		A	A	A	A	A	A	A	A	A	
6	99203222	104.0866	rs4406241	0.159	B	B	B	B	B	B	B	B			B		B	B
6	99214884	104.0911	rs7762570	0.496		B	B		B		B	B		B	B			
6	99218958	104.1162	rs4839976	0.473		B	B		B	B	B	B			B		B	
6	99219522	104.1599	rs4398748	0.92	A	A	A	A	A	A	A	A	A	A	A	A		A
6	99221669	104.1936	rs9385493	0.396	B	B	B		B	B	B	B	B		B	B	B	
6	99226729	104.1955	rs10499023	0.719	B	B										B	B	
6	99229574	104.1966	rs4839977	0.243	B	B		B		B			B	B		B	B	B
6	99230880	104.197	rs4555920	0.566	A	A	A		A	A	A	A			A	A	A	
6	99235909	104.2181	rs9375642	0.761	A	A	A	A	A					A		A	A	A
6	99236649	104.2193	rs9402154	0.429	A	A	A		A					A		A	A	
6	99237315	104.22	rs9388719	0.668	A	A	A		A	A	A	A	A	A	A	A	A	
6	99237479	104.2201	rs12174549	0.838	A	A	A	A	A					A		A	A	A
6	99242592	104.2211	rs7762652	0.661	A	A	A		A	A	A	A	A	A	A	A	A	
6	99250916	104.2243	rs9385517	0.204	B	B	B	B	B					B		B	B	B
6	99251754	104.2245	rs4839986	0.633	A	A	A		A	A	A	A	A	A	A	A	A	
6	99258445	104.2251	rs6569636	0.327	B	B	B		B	B	B	B	B	B	B	B	B	
6	99270438	104.2269	rs9375688	0.393	A	A	A		A					A		A	A	
6	99270533	104.227	rs9375689	0.673	A	A	A		A	A	A	A	A	A	A	A	A	
6	99275290	104.2312	rs2444935	0.164	B	B	B	B	B					B		B	B	B
6	99277867	104.2334	rs1869641	0.407	B	B	B		B	B	B	B	B	B	B	B	B	
6	99279449	104.234	rs1883306	0.956	A	A	A	A	A		A	A	A	A	A	A	A	A
6	99284532	104.2365	rs3823036	0.699	B	B	B		B							B	B	
6	99290334	104.2388	rs195853	0.155	B	B	B	B	B					B		B	B	B
6	99290592	104.2389	rs195852	0.403	B	B	B		B	B	B	B	B	B	B	B	B	
6	99294322	104.2405	rs195851	0.531	A	A	A		A	A	A	A	A	A	A	A	A	
6	99309057	104.2804	rs174447	0.058	B	B	B	B	B	B	B	B	B		B	B	B	B
6	99320715	104.2812	rs9388789	0.09	B	B	B	B	B	B	B	B		B	B	B	B	B
6	99323424	104.2812	rs11537982	0.062	B	B	B	B	B	B	B	B	B	B	B	B	B	B
6	99326490	104.2812	rs9375728	0.885	A	A	A	A	A	A	A	A		A	A	A	A	A
6	99328730	104.284	rs195831	0.637	B	B	B		B	B	B	B			B		B	
6	99336279	104.284	rs10484609	0.85	A	A	A	A	A	A	A	A		A	A	A	A	A
6	99346145	104.2845	rs7739884	0.429	B	B	B		B	B	B	B	B	B	B		B	
6	99374400	104.2862	rs1011676	0.071	B	B	B	B	B	B	B	B	B	B	B	B	B	B
6	99431200	104.2903	rs17058986	1	A	A	A	A	A	A	A	A	A	A	A	A	A	A
6	99432747	104.2906	rs196959	0.377	A	A	A		A	A	A	A			A		A	
6	99433614	104.2908	rs196960	0.881	A	A	A	A	A	A	A	A	A		A	A	A	A
6	99437566	104.2928	rs2747734	0.473	B	B	B		B	B	B	B		B	B	B	B	
6	99440369	104.2932	rs2572109	0.093	B	B	B	B	B	B	B	B		B	B	B	B	B
6	99441083	104.2937	rs12200990	0.404	B	B	B		B	B	B	B	B	B	B	B	B	
6	99450081	104.2967	rs2180046	0.21	B		B	B	B				B	B		B	B	B
6	99462075	104.2976	rs2747739	0.296	A		A		A				A				A	
6	99462324	104.2977	rs9402354	0.81	B		B		B								B	
6	99463771	104.298	rs9375844	0.265	A		A		A								A	
6	99464542	104.2983	rs2207446	0.258	B		B	B	B				B			B	B	B
6	99467661	104.2997	rs12173555	0.102	B	B	B	B	B	B	B	B		B	B	B	B	B
6	99468942	104.3004	rs9388950	0.336	A		A		A					A			A	
6	99473410	104.3017	rs2092772	0.354	B	B	B		B	B	B	B	B	B	B		B	
6	99478174	104.3023	rs12201236	0.308	B	B	B		B	B	B	B	B	B	B		B	
6	99478304	104.3024	rs4839737	0.062	B	B	B	B	B	B	B	B	B		B	B	B	B
6	99479842	104.3085	rs2747748	0.73	A		A	A	A				A	A		A	A	A
6	99480633	104.3095	rs7756447	0.478	A	A				A	A	A			A		A	
6	99485266	104.3133	rs11756151	0.066	B	B	B	B	B	B	B	B	B	B	B	B	B	B
6	99498599	104.3187	rs12173832	0.192	A												A	
6	99500963	104.3191	rs2144241	0.496	A		A		A				A	A			A	
6	99501709	104.3193	rs6916751	0.304	A		A		A				A				A	
6	99504377	104.32	rs9493282	0.174	B	B	B	B	B	B	B	B	B		B	B	B	B
6	99515134	104.325	rs12660289	0.265	A		A		A				A				A	
6	99521031	104.3263	rs2207445	0.277	B		B	B	B				B	B		B	B	B
6	99524062	104.3271	rs7764372	0.673	B		B		B				B				B	
6	99524281	104.3272	rs9375894	0.071	B	B		B		B	B	B		B	B	B	B	B
6	99531172	104.3289	rs12212298	0.668	A	A	A		A	A	A	A	A		A		A	
6	99531774	104.3289	rs9385615	0.938	A	A		A		A	A	A		A	A	A	A	A
6	99538132	104.3462	rs2572098	0.915	A		A	A	A		A	A	A	A	A	A	A	A
6	99539153	104.3465	rs9402444	0.916	A	A		A		A	A	A		A	A	A	A	A
6	99550464	104.3503	rs6902772	0.544	B			B					B				B	
6	99551169	104.3504	rs6940049	0.615	A			A					A				A	
6	99551765	104.3506	rs3860236	0.133	B	B	B	B	B	B	B	B	B		B	B	B	B
6	99551970	104.3506	rs10457592	0.196	B	B	B	B	B	B			B				B	
6	99552868	104.3512	rs12208335	0.839	A	A	A	A	A	A	A	A	A	A	A	A	A	A
6	99557664	104.3539	rs6938641	0.802	A			A			A	A	A	A	A	A	A	A
6	99559773	104.354	rs1081025	0.058	B	B	B	B	B	B	B	B		B	B	B	B	B
6	99565215	104.3544	rs12526079	0.394	A			A									A	
6	99569709	104.3553	rs7769941	0.894	A			A			A	A	A	A	A	A	A	A
6	99574363	104.3568	rs2388839	0.08	B	B	B	B	B	B	B	B	B		B	B	B	B

Chromosome	Physical position (hg19)	Genetic map	rs number	Allele A frequency	Family 11 (GC15416)	Family 12 (GC3722)					Family 13 (GC17225)							
					III:3	III:11	IV:2	II:7	IV:6	IV:13	IV:57	IV:58	II:9	III:27	IV:56	III:6	IV:13	III:14
6	99575661	104.3569	rs12111251	0.004	B	B	B	B	B	B	B	B	B	B	B	B	B	B
6	99575874	104.3569	rs4454147	0.558	A			A			A	A			A		A	A
6	99583773	104.357	rs2029964	0.442	A			A					A				A	
6	99584658	104.357	rs728758	0.164	B	B	B	B	B	B			B	B			B	
6	99592404	104.3637	rs11154718	0.389	B			B			B	B	B		B	B	B	B
6	99593258	104.3642	rs12190591	0.898	A	A	A	A	A	A	A	A	A	A	A	A	A	A
6	99600083	104.3678	rs9321394	0.254	B			B			B	B		B	B	B	B	B
6	99610074	104.3872	rs4839999	0.115	B		B	B	B	B	B	B		B	B	B	B	B
6	99613136	104.3888	rs12206927	0.478	B			B			B	B			B	B	B	B
6	99629252	104.3929	rs12110525	0.255	A												A	
6	99630038	104.393	rs9373057	0.288	B	B		B			B	B	B	B	B	B	B	B
6	99633011	104.3931	rs9399068	0.279	B	B	B		B	B			B	B			B	
6	99637123	104.3941	rs9375977	0.721	A	A	A		A	A			A	A			A	
6	99640610	104.3942	rs7745052	0.181	B		B	B	B	B	B	B			B	B	B	B
6	99641248	104.3942	rs1496971	0.281	B	B	B		B	B			B	B			B	
6	99646240	104.3944	rs1908804	1	A	A	A	A	A	A	A	A	A	A	A	A	A	A
6	99659802	104.3948	rs7767885	0.531	A			A			A	A			A	A	A	A
6	99662681	104.3963	rs7769752	0.332	B	B				B			B	B			B	
6	99662743	104.3964	rs17059246	0.575	B	B							B	B			B	
6	99664294	104.3968	rs9389116	0.243	B	B	B	B	B		B	B	B	B	B	B	B	B
6	99665178	104.3971	rs4458696	0.054	B	B		B		B	B	B	B	B	B	B	B	B
6	99669676	104.4173	rs9373072	0.606	A	A		A			A	A	A		A		A	
6	99672014	104.4199	rs2132683	0.69	B	B							B					
6	99673152	104.4299	rs9375997	0.655	A	A		A			A	A	A		A		A	
6	99673466	104.4396	rs9402556	0.164	B	B	B	B	B				B	B			B	
6	99675908	104.5062	rs12528619	0.243	B	B	B	B	B	B	B	B	B	B	B	B		B
6	99678424	104.5068	rs1566116	0.364	B		B	B	B		B	B			B		B	
6	99679578	104.507	rs4840017	0.538	A			A			A	A			A		A	
6	99679902	104.5071	rs9402564	0.094	A													
6	99683181	104.5081	rs10155713	0	B	B	B	B	B	B	B	B	B	B	B	B	B	B
6	99683802	104.5084	rs2029965	0.54	A			A			A	A			A		A	
6	99690090	104.5117	rs9376014	0.757	A	A	A		A	A			A	A		A	A	A
6	99690449	104.5118	rs12193060	0.792	A	A				A			A	A		A	A	A
6	99694494	104.514	rs1874538	0.796	A	A	A		A	A			A	A		A	A	A
6	99695108	104.5143	rs6904604	0	B	B	B	B	B	B	B	B	B	B	B	B	B	B
6	99711169	104.5272	rs9493928	0	B	B	B	B	B	B	B	B	B	B	B	B	B	B
6	99717329	104.5272	rs9483707	0.673	A	A		A		A	A	A		A	A			A
6	99717914	104.5272	rs13219146	0.823	A	A	A	A	A		A	A	A	A	A	A	A	
6	99721049	104.5274	rs1045728	0.531	A	A		A		A	A	A		A	A			A
6	99724713	104.5276	rs910034	0.688	A	A	A	A	A	A	A	A		A	A			A
6	99727653	104.528	rs12660321	0.133	B	B	B	B	B	B	B	B	B	B	B	B	B	B
6	99728442	104.5292	rs1496979	0.615	A	A	A		A				A			A	A	
6	99729901	104.5298	rs6933093	0.535	A	A		A		A	A	A		A	A			A
6	99733939	104.5299	rs9373105	0.858	B	B												
6	99734185	104.5299	rs1496980	0.854	A	A	A	A	A	A	A	A		A	A		A	A
6	99740492	104.53	rs12207550	0.022	B	B		B		B	B	B	B	B	B	B	B	B
6	99744107	104.5321	rs221582	0.668	A	A		A		A	A	A		A	A			A
6	99747633	104.5339	rs221578	0.814	A	A		A		A	A	A	A	A	A	A		A
6	99753499	104.5362	rs221530	0.783	A	A		A		A	A	A	A	A	A	A		A
6	99763266	104.5472	rs6913076	0.19	B	B	B	B	B	B	B	B	B	B	B	B		B
6	99768894	104.5482	rs182613	0.623	A			A		A	A	A		A	A		A	A
6	99771540	104.5487	rs221527	0.146	B	B	B	B	B	B	B	B	B	B	B	B		B
6	99772374	104.549	rs13206094	0.758	B					B								B
6	99773417	104.551	rs4840031	0.102	B		B	B	B	B	B	B	B	B	B	B	B	B
6	99776986	104.5526	rs6922449	0.527	B			B		B	B	B		B	B			B
6	99781218	104.5532	rs11757364	0.451	A			A		A	A	A		A	A			A
6	99782879	104.5535	rs17059400	0.885	A	A	A	A	A	A	A	A	A	A	A	A	A	A
6	99793615	104.5557	rs11963108	0.332	A			A			A	A		A	A			
6	99801698	104.5576	rs9402701	0.173	B	B	B	B	B		B	B	B	B	B	B	B	
6	99803269	104.5576	rs12198238	0.633	A			A		A	A	A		A	A		A	A
6	99804157	104.5582	rs13194648	0.146	B	B	B	B	B		B	B	B	B	B	B	B	
6	99806408	104.5584	rs17059457	0.908	A		A	A	A	A	A	A	A	A		A	A	A
6	99814530	104.5602	rs4839747	0	B	B	B	B	B	B	B	B	B	B	B	B	B	B
6	99819379	104.5612	rs6925344	0.151	B	B	B	B	B		B	B	B	B	B	B	B	
6	99826265	104.5623	rs12193590	0.704	A			A		A	A	A		A	A		A	A
6	99827314	104.5626	rs9402716	0.31	B	B	B	B	B		B	B	B	B	B	B		
6	99828941	104.563	rs4840038	0.308	B			B		B	B	B		B	B		B	B
6	99836954	104.5765	rs11154812	0.097	B	B	B	B	B	B	B	B	B	B	B	B	B	B
6	99842056	104.577	rs9376137	0.252	B	B	B	B	B	B			B			B	B	B
6	99842138	104.5771	rs9376138	0.345	A						A	A		A	A			
6	99847168	104.5771	rs11961608	1	A	A	A	A	A	A	A	A	A	A	A	A	A	A
6	99847260	104.5771	rs4144165	0.531	A						A	A		A	A		A	
6	99851977	104.5777	rs3811072	0.509	A						A	A		A	A		A	
6	99852267	104.5777	rs11154824	0.839	A	A	A	A	A	A	A	A	A	A	A	A		A
6	99857773	104.5777	rs9321534	0.5	A						A	A		A	A		A	
6	99869689	104.5782	rs4840039	0.008	B	B	B	B	B	B	B	B	B	B	B	B	B	B
6	99871010	104.5782	rs12198321	0.509	A						A	A		A	A		A	
6	99873534	104.5789	rs4351270	0.161	B	B	B	B	B	B	B	B	B	B	B	B		B
6	99877219	104.5813	rs6923983	0.513	B			B		B	B	B		B	B			B
6	99880380	104.5813	rs1134718	0.947	A	A	A	A	A	A	A	A	A	A	A	A	A	A
6	99880572	104.5813	rs12214037	0.812	A	A	A	A	A	A	A	A	A	A	A	A		A
6	99882563	104.5813	rs17785525	0.142	B	B	B		B		B	B	B	B	B	B	B	
6	99883137	104.5813	rs6570064	0.513	B			B		B	B	B		B	B			B
6	99883694	104.5813	rs9402791	0.754	A	A	A	A	A	A			A			A	A	A
6	99883704	104.5813	rs6570065	0	B	B	B	B	B	B	B	B	B	B	B	B	B	B
6	99889915	104.5826	rs4839748	0.354	B			B		B	B	B		B	B		B	B
6	99893527	104.5873	rs9494471	0.054	B	B	B	B	B		B	B	B	B	B	B	B	B
6	99893878	104.5886	rs12203426	0.805	A	A	A	A	A	A	A	A	A	A	A	A		A
6	99893938	104.5887	rs4504482	0.664	A		A		A	A			A			A	A	
6	99901608	104.5901	rs10155760	0	B	B	B	B	B	B	B	B	B	B	B	B	B	B
6	99905558	104.5905	rs6916603	0.659	A	A		A		A	A	A		A	A		A	A
6	99916182	104.5919	rs10457650	0.571	B	B		B			B	B		B	B			B
6	99921822	104.593	rs7745012	0.231	B	B	B	B	B	B	B	B	B	B	B	B		B
6	99933680	104.5946	rs9483935	0.1	A													
6	99934446	104.5946	rs6918880	0.702	A		A		A	A			A			A	A	
6	99939045	104.595	rs12717185	0.836	B					B								
6	99964012	104.6003	rs2209157	0.204	B	B	B	B	B	B	B	B	B	B	B	B		B
6	99971429	104.6019	rs9402863	0.394	A					A				A			A	
6	99973893	104.6045	rs10223892	0.217	A	A				A				A				
6	99974948	104.6072	rs17224695	0.781	A	A	A	A	A	A	A	A	A	A	A			A
6	99980252	104.6088	rs2057517	0.889	B													
6	99980522	104.6089	rs2057518	0.808	A	A	A	A	A	A	A	A	A	A	A	A		A
6	99986195	104.6097	rs7754710	0.616	A	A	A	A	A	A	A	A	A		A			A
6	99990856	104.6151	rs1054227	0.832	A	A	A	A	A	A	A	A	A		A		A	A
6	99993268	104.6151	rs2296154	0.196	B	B	B	B	B	B	B	B	B		B		B	B
6	99995320	104.6151	rs13205324	0.177	A	A				A	A	A			A			
6	100005775	104.6152	rs543967	0.407	B	B				B	B	B		B	B	B	B	
6	100022125	104.6162	rs1590359	0.208	A	A				A	A	A			A			
6	100031037	104.6174	rs514769	0.576	A	A	A		A	A	A	A			A	A	A	
6	100046845	104.6227	rs1552855	0.584	A	A	A		A	A	A	A			A	A	A	
6	100046933	104.6229	rs9484083	0.146	B	B		B		B	B	B	B	B	B		B	B
6	100053626	104.6362	rs7741279	0.115	B	B		B		B	B	B	B	B	B		B	B
6	100060761	104.6388	rs330843	0.235	B	B	B		B	B	B	B	B	B	B	B	B	
6	100062766	104.6392	rs3734346	0.942	A	A		A		A	A	A	A	A	A	A	A	A
6	100063243	104.6392	rs330844	0.208	B	B	B	B	B	B	B	B	B	B	B	B		B
6	100081441	104.6424	rs6927488	0.088	B	B	B	B	B	B	B	B	B	B	B		B	B
6	100082983	104.6427	rs594231	0.23	B	B	B		B	B	B	B	B	B	B	B	B	
6	100088902	104.6439	rs9402954	0.943	A	A		A		A	A	A		A	A	A	A	A
6	100092390	104.6441	rs1339203	0.739	B									B				
6	100099703	104.6445	rs546567	0.323	B	B	B		B	B	B	B	B	B	B		B	

Chromosome	Physical position (hg19)	Genetic map	rs number	Allele A frequency	Family 11 (GC15416)	Family 12 (GC3722)					Family 13 (GC17225)							
					III:3	III:11	IV:2	II:7	IV:6	IV:13	IV:57	IV:58	II:9	III:27	IV:56	III:6	IV:13	III:14
6	100108500	104.6449	rs13193313	0.9	A		A	A	A				A	A		A	A	A
6	100116092	104.6739	rs9321659	0.116	B	B		B		B	B	B	B	B	B	B	B	B
6	100125119	104.6744	rs650783	0.792	A	A	A	A	A	A	A	A	A	A	A	A		A
6	100129570	104.6745	rs9373217	0.044	B	B	B	B	B	B	B	B	B	B	B	B	B	B
6	100129959	104.6745	rs503649	0.77	A	A	A		A	A	A	A		A	A	A	A	
6	100130991	104.6745	rs472977	0.199	B	B	B		B	B	B	B		B	B	B	B	
6	100131219	104.6745	rs9495145	0.049	B	B	B	B	B	B	B	B	B	B	B	B	B	B
6	100132920	104.6753	rs9376355	0.954	A	A	A	A	A	A	A	A	A	A	A	A	A	A
6	100161035	104.6765	rs12661094	0.54	B			B					B	B				B
6	100172277	104.6779	rs12211649	0.066	B	B		B		B	B	B	B	B	B	B	B	B
6	100173173	104.6779	rs4839755	0.212	B		B	B	B				B	B			B	B
6	100173832	104.6779	rs9389645	0.681	A	A		A		A	A	A	A	A	A	A		A
6	100174464	104.678	rs6916754	0.044	B	B	B	B	B	B	B	B	B	B	B	B	B	B
6	100185261	104.6783	rs6908196	0.575	B			B					B	B				B
6	100187748	104.6827	rs12527523	0.274	B	B		B		B	B	B	B	B	B	B		B
6	100192215	104.6875	rs12525414	0.738	A	A		A		A	A	A	A	A	A	A		A
6	100195578	104.6879	rs17826560	0.765	A		A	A	A				A	A			A	A
6	100197285	104.6881	rs7742890	0.73	A	A	A		A	A	A	A		A	A	A	A	
6	100197712	104.6883	rs7356874	0.058	B	B	B	B	B	B	B	B	B	B	B	B	B	B
6	100206681	104.6904	rs10485226	0.226	A									A				
6	100207327	104.6905	rs10485227	0.54	B	B				B	B	B		B	B	B		
6	100207838	104.6906	rs4388294	0.721	A	A	A		A	A	A	A		A	A	A	A	
6	100208624	104.6908	rs17059765	0.973	A	A	A	A	A	A	A	A	A	A	A	A	A	A
6	100213515	104.6975	rs4431442	0.677	B									B				
6	100228256	104.7098	rs9403091	0.212	B	B	B	B	B	B	B	B	B		B		B	B
6	100231618	104.7102	rs9376435	0.447	B									B		B	B	
6	100233278	104.7105	rs9385869	0.238										A				
6	100233870	104.7107	rs9399284	0.508										A		A	A	
6	100236473	104.7122	rs12663112	0.823		A	A	A	A	A	A	A	A	A	A		A	A
6	100236866	104.7124	rs4596491	0.102		B	B	B	B	B	B	B	B	B	B	B	B	B
6	100237464	104.7128	rs12198721	0.508	A	A	A		A		A	A		A	A	A		
6	100237880	104.7135	rs6936455	0.2	B	B	B		B		B	B		B	B	B	B	
6	100240267	104.7143	rs9403103	0.235	B	B	B		B		B	B		B	B	B	B	
6	100242158	104.7145	rs6929006	0.513		B	B		B		B	B		B	B		B	
6	100242366	104.7145	rs6929428	0.336	B	B	B		B		B	B		B	B	B	B	
6	100243033	104.7146	rs17236659	0.783				A		A			A	A		A	A	A
6	100243997	104.716	rs9376456	0.412	B									B			B	
6	100247048	104.7176	rs6931227	0.177										A				
6	100249106	104.7185	rs7763983	0.735		B	B		B		B	B		B	B			
6	100250740	104.7245	rs17059858	0.211										A				
6	100251499	104.7264	rs9484314	0.198										A				
6	100252886	104.7267	rs17059867	0	B	B	B	B	B	B	B	B	B	B	B	B	B	B
6	100255471	104.7271	rs9373250	0.27	B	B	B		B		B	B		B	B	B	B	
6	100257066	104.7276	rs12216503	0.111	B			B		B			B	B		B	B	B
6	100257668	104.7279	rs4495279	0.226	B	B	B	B	B	B	B	B	B	B	B	B		B
6	100265121	104.7348	rs6934621	0.201		B	B	B	B	B	B	B	B	B	B		B	B
6	100266123	104.7357	rs7760502	0.665		A	A	A	A	A	A	A	A		A		A	A
6	100268849	104.7382	rs17059881	0.872		A	A	A	A	A	A	A	A	A	A	A	A	A
6	100277840	104.7428	rs13204333	0.254	B	B		B		B	B	B	B		B		B	B
6	100279956	104.7518	rs9403141	0.217		B	B	B	B	B			B	B			B	B
6	100280032	104.7521	rs9385894	0.278			B	B	B	B			B	B			B	B
6	100281817	104.7551	rs7453413	0.403	A	A		A			A	A			A	A		
6	100282075	104.7553	rs3922542	0.617	B	B		B			B	B			B	B		
6	100286127	104.7628	rs9321763	0.476				B		B	B	B		B	B	B		B
6	100288317	104.8184	rs4370372	0.637		A		A		A				A		A	A	A
6	100289337	104.8716	rs9389752	0.31	B		B	B	B	B			B	B		B	B	
6	100292091	104.8879	rs9495653	0.929	A	A	A	A	A		A	A	A	A	A	A	A	A
6	100293531	104.8906	rs9495657	0.317	A			A		A							A	
6	100296024	104.8908	rs4990843	0.628	B			B		B							B	
6	100297481	104.8909	rs9403163	0.73	A	A	A	A	A	A	A	A	A	A	A	A	A	
6	100317146	104.8946	rs12197810	0.73	A			A		A			A				A	A
6	100319031	104.8949	rs9403180	0.375	A			A		A							A	
6	100321669	104.8957	rs2397663	0.992	A	A	A	A	A	A	A	A	A	A	A	A	A	A
6	100321734	104.8957	rs2397664	0.921	A	A	A	A	A	A	A	A	A	A	A	A	A	A
6	100324464	104.8958	rs9403186	0.714	A	A	A	A	A	A	A	A		A	A	A	A	
6	100326214	104.8978	rs9495745	0.219	B			B		B			B			B	B	B
6	100328198	104.9045	rs4840097	0.642	B			B		B							B	
6	100329446	104.9064	rs9389810	0.721	A			A		A	A	A	A		A	A	A	A
6	100345238	104.9096	rs2397678	0.354	A			A		A						A	A	
6	100345403	104.9098	rs6902801	0.604	A	A	A	A	A	A				A		A	A	
6	100353268	104.9202	rs7751620	0.261	A			A									A	
6	100354935	104.9231	rs9403208	0.451	B	B	B	B	B					B			B	
6	100366635	104.9245	rs4560657	0.212	A			A									A	
6	100369284	104.9251	rs13206575	0.723	B			B									B	
6	100391872	104.9275	rs4559096	0.31	B			B		B	B	B	B		B	B	B	B
6	100392532	104.9276	rs12215494	0.308	B			B		B	B	B	B		B	B	B	B
6	100393761	104.9277	rs13195863	0.243	A			A									A	
6	100397526	104.9282	rs10499026	0.217	A			A									A	
6	100403514	104.9289	rs4840106	0.304	B	B	B	B	B	B				B		B	B	B
6	100411417	104.9299	rs7758072	0	B	B	B	B	B	B	B	B	B	B	B	B	B	B
6	100427015	104.9418	rs7739904	0.365	B	B	B	B	B				B				B	
6	100428029	104.9567	rs9496070	0.5		B		B		B			B			B	B	
6	100428313	104.9588	rs13212643	0.419		A		A					A				A	
6	100430062	104.9612	rs12203515	0.208		B		B		B	B	B	B	B	B	B	B	B
6	100430803	104.9623	rs9496085	0.373	B	B	B	B	B				B				B	
6	100431321	104.9629	rs9389934	0.616	A	A	A	A	A				A				A	
6	100439009	104.9681	rs2001456	0.226	B		B	B	B	B	B	B		B	B	B	B	B
6	100442268	104.9706	rs6925272	0.602	B		B	B	B								B	
6	100442554	104.9708	rs9969034	0.009	B	B	B	B	B	B	B	B	B		B	B	B	B
6	100445429	104.9741	rs2397694	0.987	A	A	A	A	A	A	A	A	A	A	A	A	A	A
6	100446900	104.9911	rs9399386	0.185	B		B	B	B	B	B	B		B	B		B	B
6	100448788	105.0153	rs3763374	0.093	B	B	B	B	B	B	B	B	B	B	B	B	B	B
6	100449301	105.022	rs9385975	0.111	B	B	B	B	B	B	B	B	B	B	B	B	B	B
6	100457411	105.025	rs9389951	0.473	B						B	B			B		B	B
6	100469529	105.0296	rs9484578	0.95	A	A	A	A	A		A	A	A	A	A		A	A
6	100480906	105.0316	rs4240585	0.315	B					B	B	B			B	B	B	B
6	100482016	105.0317	rs6925134	0.097	B	B	B	B	B	B			B	B		B	B	
6	100492824	105.033	rs12202476	0.089	B	B	B	B	B	B	B	B	B	B	B	B	B	B
6	100494186	105.0332	rs3890820	0.146	B	B	B	B	B	B	B	B	B	B	B	B	B	B
6	100510685	105.0371	rs6903499	0.066	B	B	B	B	B		B	B	B	B	B		B	B
6	100514432	105.0376	rs11155243	0.173	B	B	B	B	B		B	B	B	B	B		B	B
6	100521649	105.0385	rs6938191	0.593	B													
6	100523912	105.039	rs4144262	0.102	B	B	B	B	B	B	B	B	B	B	B	B	B	B
6	100534262	105.0457	rs2474265	0.062	B	B	B	B	B		B	B	B	B	B		B	B
6	100539396	105.0464	rs6910833	0.576	B													
6	100548788	105.047	rs17060130	0.888	A	A	A	A	A	A			A	A		A	A	
6	100554149	105.0484	rs12195124	0.094	B	B	B	B	B	B	B	B	B	B	B	B	B	B
6	100560563	105.0499	rs9386011	0.69	A					A	A	A			A	A		A
6	100562360	105.0512	rs2474258	0.088	B	B	B	B	B				B	B			B	
6	100574090	105.0515	rs9969059	0.092	B	B	B	B	B	B	B	B	B	B	B	B	B	B
6	100575272	105.0521	rs932511	0.482	B					B	B	B			B			B
6	100583430	105.0525	rs4839772	0.389	B					B	B	B			B	B		B
6	100592593	105.1184	rs9390065	0.336	B	B	B	B	B		B	B	B	B	B	B	B	B
6	100592868	105.133	rs2474254	0.748	A	A	A		A	A	A	A	A	A	A	A		A
6	100593022	105.1412	rs11155282	0.603							B	B	B		B		B	B
6	100600097	105.2122	rs17789218	0.783	B								B			B	B	
6	100601918	105.2129	rs2474251	0.929	A	A	A	A	A				A	A		A	A	A
6	100602931	105.2131	rs2503095	0.131	B	B		B		B	B	B	B	B	B	B	B	B
6	100603029	105.2131	rs761714	0.2	B	B	B		B	B	B	B	B	B	B	B	B	
6	100606568	105.2132	rs2503097	0.81	A	A		A		A	A	A	A	A	A	A	A	A

Chromosome	Physical position (hg19)	Genetic map	rs number	Allele A frequency	Family 11 (GC15416)	Family 12 (GC3722)						Family 13 (GC17225)							
					III:3	III:11	IV:2	II:7	IV:6	IV:13	IV:57	IV:58	II:9	III:27	IV:56	III:6	IV:13	III:14
6	100612700	105.2132	rs17789452	0.345	B		B	B	B	B	B	B	B		B	B	B	B
6	100618009	105.2148	rs6916538	0.772	A	A		A					A	A		A	A	A
6	100626148	105.2171	rs761711	0.465	A			A		A	A	A	A		A	A	A	A
6	100627435	105.2176	rs13205707	0.381	B		B	B	B	B	B	B	B		B	B	B	B
6	100631103	105.3208	rs7741147	0.619	A	A	A		A		A	A	A		A	A	A	
6	100633338	105.3305	rs12201659	0.398		B	B	B	B	B	B	B		B	B			B
6	100634009	105.3324	rs6899958	0.193	B	B	B	B	B		B	B	B	B	B	B	B	B
6	100636619	105.3449	rs7754315	0.429		A	A		A	A	A	A		A	A			A
6	100642867	105.4553	rs6570574	0.221	B	B	B	B	B	B	B	B	B	B	B	B	B	
6	100650387	105.4555	rs9496769	0.246						B			B			B		B
6	100661336	105.4569	rs9484823	0.854	A					A			A	A		A		A
6	100661466	105.457	rs9399465	0.04	B	B	B	B	B	B	B	B	B	B	B	B	B	B
6	100668277	105.46	rs9390146	0.097		B	B	B	B	B	B	B	B		B	B	B	B
6	100674018	105.463	rs6928245	0.332		B	B	B	B	B	B	B	B		B	B	B	
6	100674799	105.4634	rs7749433	0.761	A	A	A	A	A	A	A	A	A	A	A	A	A	
6	100676593	105.4642	rs730223	0.584						A			A			A		
6	100680565	105.4679	rs2073265	0.384	B					B			B	B		B		
6	100693008	105.4694	rs4140483	0.133	B					B			B	B		B		B
6	100698564	105.4701	rs9390162	0.827	A					A			A	A		A		A
6	100706809	105.471	rs12175103	0.406	B					B			B	B		B		
6	100707145	105.4711	rs6908458	0.754	A	A	A	A	A	A	A	A	A	A	A	A	A	
6	100722593	105.4729	rs7750764	0.092	B	B	B	B	B	B	B	B	B	B	B	B	B	B
6	100734930	105.4735	rs9399482	0.404	B					B			B	B		B		
6	100743929	105.4776	rs9376845	0.057	B	B	B	B	B	B	B	B	B	B	B	B	B	B
6	100744281	105.4776	rs10499030	0.261	B	B	B	B	B	B	B	B	B	B	B	B	B	
6	100752002	105.4776	rs9390223	0.757	A	A	A	A	A	A	A	A	A	A	A	A	A	
6	100758142	105.4841	rs11752981	0.808	A	A	A	A	A	A	A	A	A	A	A	A	A	
6	100760042	105.5016	rs9376867	0.288	B	B	B	B	B	B	B	B	B	B	B	B	B	
6	100760170	105.5033	rs13196561	0.204	B	B	B	B	B	B	B	B	B	B	B	B	B	
6	100760409	105.5064	rs3957418	0.376	A								A			A		
6	100763213	105.5223	rs924974	0.35	B	B	B	B	B	B	B	B	B	B	B	B		
6	100769678	105.5227	rs10046239	0.912	A	A	A	A	A	A	A	A	A	A	A	A	A	A
6	100773433	105.5231	rs1506075	0.903	A					A			A	A		A	A	A
6	100776957	105.5235	rs2658132	0.008	B	B	B	B	B	B	B	B	B	B	B	B	B	B
6	100779276	105.524	rs2841300	0.357	B	B	B	B	B	B	B	B	B	B	B	B		
6	100779692	105.5244	rs9390276	0.933	A	A	A	A	A	A	A	A	A	A	A	A	A	A
6	100781576	105.5246	rs2658128	0	B	B	B	B	B	B	B	B	B	B	B	B	B	B
6	100785588	105.5251	rs2841296	0.867	A	A	A	A	A	A	A	A	A	A	A	A		A
6	100791267	105.5261	rs1395118	0.783	A	A	A	A	A	A	A	A	A	A	A	A	A	
6	100793016	105.5274	rs7749859	0.044	B	B	B	B	B	B	B	B	B	B	B	B		B
6	100793443	105.5277	rs2841292	0.686	A	A	A	A	A	A	A	A	A	A	A	A	A	
6	100797890	105.534	rs1876155	0.159	B	B		B		B	B	B	B	B	B	B	B	B
6	100798472	105.5356	rs1506079	0.111			B		B	B			B	B		B	B	B
6	100800122	105.5371	rs2841287	0.273						B			B	B		B		B
6	100802068	105.5379	rs9403683	0.863	A	A	A	A	A	A	A	A	A	A	A	A		A
6	100810976	105.543	rs9403695	0.074	B	B	B	B	B	B	B	B	B	B	B	B	B	B
6	100811392	105.5528	rs17060420	0.916	A	A	A	A	A	A	A	A	A	A	A	A	A	A
6	100811533	105.5562	rs17792983	0.252	B	B	B	B	B	B	B	B	B	B	B	B	B	
6	100813188	105.5838	rs1506084	0.35		B		B		B			B	B		B	B	
6	100816614	105.5848	rs13213077	0.231		B		B		B			B	B		B	B	
6	100818915	105.5855	rs9376940	0.252		B		B		B			B	B		B	B	
6	100827450	105.5904	rs9403718	0.761		A		A		A			A	A		A	A	A
6	100827834	105.5905	rs17060460	0.759		A		A		A			A	A		A	A	A
6	100829135	105.5906	rs453530	0.036	B	B	B	B	B	B	B	B	B	B	B	B		B
6	100831997	105.5907	rs9403732	0.146		B		B		B			B	B		B	B	B
6	100836784	105.591	rs13201004	0.106	B	B	B	B	B	B	B	B	B	B	B	B	B	
6	100857869	105.5953	rs7747189	0.248		B		B		B			B	B		B	B	
6	100859159	105.5956	rs718268	0.754		A		A		A			A	A		A	A	
6	100859947	105.5958	rs17060530	0.685		A		A		A			A	A		A	A	
6	100862051	105.5964	rs17060534	0.093	B	B	B	B	B	B	B	B	B	B	B	B	B	B
6	100868779	105.5986	rs3734354	0.146	B	B		B		B			B	B		B	B	B
6	100872787	105.6067	rs714378	0.204	B	B	B	B	B	B	B	B	B	B	B	B	B	
6	100875516	105.631	rs3778033	0.783		A		A		A			A			A	A	A
6	100878267	105.7577	rs3798498	0.549	A	A	A	A	A		A	A	A		A	A		
6	100879651	105.7594	rs241816	0.584	A	A	A	A	A		A	A	A	A	A	A		
6	100880696	105.7602	rs1847912	0.712	A	A	A	A	A	A	A	A	A		A	A		
6	100888625	105.7632	rs3798492	0.319	B	B	B	B	B	B	B	B	B	B	B	B		
6	100894587	105.7639	rs1857859	0.321	B	B	B	B	B	B	B	B	B	B	B	B		
6	100895344	105.7639	rs397662	0.836	A	A	A	A	A		A	A	A	A	A	A	A	A
6	100897259	105.7644	rs438766	0.009	B	B	B	B	B	B	B	B	B	B	B	B	B	B
6	100906504	105.7667	rs3798485	0	B	B	B	B	B	B	B	B	B	B	B	B	B	B
6	100910798	105.7681	rs241819	0.496	A	A	A	A	A		A	A		A	A	A		
6	100913553	105.7683	rs6928357	0.025	B	B	B	B	B	B	B	B	B	B	B	B	B	B
6	100932409	105.7743	rs1972281	0.758	A	A	A	A	A		A	A		A	A	A	A	A
6	100936361	105.7746	rs6570772	0.173	B	B	B	B	B		B	B		B	B	B	B	B
6	100936628	105.7746	rs10457800	0.465	A	A	A	A	A		A	A		A	A	A		
6	100942138	105.7766	rs7764271	0.023	B	B	B	B	B	B	B	B	B	B	B	B	B	B
6	100949822	105.7771	rs7751526	0.496	B	B	B	B	B		B	B		B	B	B		
6	100953148	105.7805	rs240764	0.403	A	A	A	A	A		A	A		A	A	A		
6	100954036	105.7828	rs240766	0.478	A	A	A	A	A		A	A	A	A	A	A		
6	100955752	105.7846	rs12374612	0.389	A	A	A	A	A		A	A		A	A	A		
6	100957344	105.7848	rs240768	0.938	A	A	A	A	A	A	A	A		A	A	A	A	A
6	100961198	105.7853	rs401864	0.92	A	A	A	A	A	A	A	A		A	A	A	A	A
6	100966006	105.7859	rs3213542	0.072	B	B	B	B	B	B	B	B		B	B	B	B	B
6	100999211	105.7861	rs17060716	0	B	B	B	B	B	B	B	B	B	B	B	B	B	B
6	101027440	105.7878	rs6931919	0.944	A	A	A	A	A	A	A	A		A	A	A	A	A
6	101039215	105.7879	rs17245314	0.707	A	A	A	A	A	A	A	A	A	A	A	A		
6	101039685	105.7879	rs7741503	0.004	B	B	B	B	B	B	B	B	B	B	B	B	B	B
6	101049108	105.7889	rs6927531	0.023	B	B	B	B	B	B	B	B	B	B	B	B	B	B
6	101072307	105.7902	rs240150	0.577	B	B	B	B	B		B	B		B	B	B		
6	101076992	105.7904	rs17246013	0.992	A	A	A	A	A	A	A	A	A	A	A	A	A	A
6	101091434	105.7912	rs1392969	0.973	A	A	A	A	A	A	A	A	A	A	A	A	A	A
6	101094554	105.7913	rs239239	0.398	A	A	A	A	A		A	A		A	A	A		
6	101109175	105.7918	rs17306522	0.111	A			A			A	A			A			
6	101147095	105.7977	rs12201157	0.089	B	B	B	B	B	B	B	B	B	B	B		B	B
6	101149540	105.7978	rs9386233	0.585	B	B	B	B	B		B	B	B	B	B	B		
6	101157916	105.7993	rs6904959	0.352	A	A	A	A	A		A	A		A	A	A		
6	101166809	105.7995	rs7748644	0.648	B	B	B	B	B		B	B		B	B	B		
6	101174245	105.801	rs12525128	0.518	B	B	B	B	B		B	B	B	B	B	B		
6	101177506	105.8017	rs6570925	1	A	A	A	A	A	A	A	A	A	A	A	A	A	A
6	101191397	105.8028	rs1039033	0.438	A	A	A	A	A		A	A	A	A	A	A		
6	101192051	105.8028	rs10485292	0.112	B	B	B	B	B	B	B	B	B	B	B		B	B
6	101196080	105.803	rs12212435	0.567	B	B	B	B	B		B	B		B	B	B		
6	101234788	105.8065	rs12664423	0.761	B			B			B	B			B	B		
6	101240763	105.8074	rs1552935	0.009	B	B	B	B	B	B	B	B	B	B	B	B	B	B
6	101247503	105.8076	rs3811078	0.553	B	B	B	B	B		B	B		B	B	B		
6	101248149	105.8084	rs12216426	0.119	B	B	B	B	B	B	B	B	B	B	B		B	B
6	101263516	105.8092	rs7740783	0.549	B	B	B	B	B		B	B		B	B	B		
6	101272419	105.8178	rs17061132	0	B	B	B	B	B	B	B	B	B	B	B	B	B	B
6	101272439	105.8178	rs846802	0.018	B	B	B	B	B	B	B	B	B	B	B	B	B	B
6	101275792	105.8265	rs705588	0.765	A			A		A	A	A			A	A	A	A
6	101295873	105.8308	rs9399696	0.938	A	A	A	A	A	A	A	A		A	A	A	A	A
6	101296389	105.8308	rs9390698	0.482	B	B	B	B	B		B	B	B	B	B	B		
6	101304411	105.832	rs9386259	0.779	A	A	A	A	A		A	A		A	A	A	A	A
6	101304829	105.832	rs705599	0.739	B			B			B	B			B	B		
6	101312424	105.832	rs998254	0.686	A	A	A	A	A	A		A	A	A	A	A		
6	101312900	105.832	rs10872662	0.217	B	B	B	B	B		B	B		B	B	B	B	B
6	101329173	105.833	rs12530388	0.423	A	A	A	A	A		A	A		A	A	A		
6	101330188	105.8338	rs4840159	0.549	B	B	B	B	B		B	B		B	B	B		
6	101344511	105.837	rs612117	0.841	A			A		A	A	A	A	A	A	A	A	A

Chromosome	Physical position (hg19)	Genetic map	rs number	Allele A frequency	Family 11 (GC15416)	Family 12 (GC3722)					Family 13 (GC17225)							
					III:3	III:11	IV:2	II:7	IV:6	IV:13	IV:57	IV:58	II:9	III:27	IV:56	III:6	IV:13	III:14
6	101351852	105.8386	rs625745	0.195	B			B		B	B	B	B		B	B	B	B
6	101351896	105.8387	rs626120	0.69	B			B			B	B	B		B	B		
6	101354510	105.8391	rs4840161	0.422	B	B	B	B	B		B	B	B	B	B	B		
6	101363967	105.8396	rs705619	0.825	A			A		A	A	A	A		A	A	A	A
6	101370840	105.8403	rs9498430	0	B	B	B	B	B	B	B	B	B	B	B	B	B	B
6	101371187	105.8404	rs10485300	0.965	A	A	A	A	A	A	A	A	A	A	A	A	A	A
6	101371644	105.8406	rs661142	0.246	A			A			A	A			A	A		
6	101371999	105.8406	rs673119	0.777	A			A		A	A	A	A		A	A	A	A
6	101380707	105.8415	rs7739574	0.758	B			B			B	B			B	B		
6	101392348	105.8437	rs595422	0.73	B			B			B	B			B	B		
6	101395407	105.8439	rs11969893	0.049	B	B	B	B	B	B	B	B		B	B	B	B	B
6	101406380	105.8456	rs6925884	0.894	A	A	A	A	A	A	A	A		A	A	A	A	A
6	101408044	105.8463	rs12195612	0.954	A	A	A	A	A	A	A	A	A	A	A		A	A
6	101409649	105.8476	rs817229	0.305	A			A			A	A	A		A	A		
6	101426759	105.8502	rs4240589	0.54	A	A	A	A	A		A	A	A	A	A	A		
6	101436242	105.8539	rs17061291	0.991	A	A	A	A	A	A	A	A	A	A	A	A	A	A
6	101449041	105.855	rs2244658	0.761	B			B			B	B			B	B		
6	101463320	105.8581	rs9404070	0.513	A	A	A	A	A		A	A		A	A	A		
6	101466600	105.8598	rs17061311	1	A	A	A	A	A	A	A	A	A	A	A	A	A	A
6	101467842	105.86	rs17061313	0	B	B	B	B	B	B	B	B	B	B	B	B	B	B
6	101468591	105.8601	rs1321204	0.925	A	A	A	A	A		A	A		A	A	A	A	A
6	101471710	105.8624	rs1575100	0.504		B				A			A	A				A
6	101481014	105.8654	rs1765075	0.169	B			B		B	B	B	B	B	B	B	B	B
6	101482379	105.8657	rs13211508	0.085			B		B	B			B	B		B		B
6	101487483	105.8674	rs12528419	0.477		B				A			A	A				A
6	101488697	105.8674	rs10499032	0.046	B	B	B	B	B	B	B	B	B	B	B	B	B	B
6	101490341	105.8675	rs9390717	0.478		B				A			A	A				A
6	101507345	105.8703	rs11155845	0.013	B	B	B	B	B	B	B	B	B	B	B	B	B	B
6	101508921	105.8705	rs9498467	0.932	A	A	A	A	A	A	A	A	A	A	A	A	A	A

Supplementary Table S2 Region with a Conserved Homozygosity Haplotype[2] (HH) at the MCDR3 locus shared by 14 affected individuals from families 2-7 with available Illumina SNP array data. The HH is a type of haplotype described by the homozygous SNPs only (all heterozygous SNPs are removed) and, therefore, can be uniquely determined on each chromosome. Genotypes are displayed vertically per each individual (A=AA, B=BB, blank cells=AB). The start and end of the shared HH are marked with a red bold line.

Chromosome	Physical position (hg19)	Genetic map	rs number	Allele A frequency	Family 2 (GC15626)		Family 3 (GC15119)				Family 4 (GC13840)			Family 5 (GC19075)			Family 6 (GC15475)	Family 7 (GC11709)
					IV:5	IV:6	IV:III	IV:II	III:1	V:1	II:1	I:1	II:2	III:4	II:3	II:2	I:1	II:1
5	4293345	10.7122	rs11742174	0.845	A	A	A	A	A	A	A	A	A	A	A	A	A	A
5	4293859	10.7127	rs7702429	0.088	B	B	B	B	B	B	B	B	B	B	B	B	B	B
5	4297423	10.7163	rs7731506	0.217	B		B	B		B	B	B	B	B	B	B	B	B
5	4303898	10.7173	rs1393106	0.385	B		B	B		B	B	B	B	B	B	B	B	B
5	4305161	10.7175	rs11134044	0.845	A	A	A	A	A	A	A	A	A	A	A	A	A	A
5	4311402	10.7206	rs155350	0.177			B	B	B	B		B			A	A		B
5	4316307	10.7218	rs261133	0.235	B		B	B		B	B	B	B	B	B	B	B	B
5	4316470	10.7218	rs11749633	0.15	B	B	B	B	B	B	B	B	B	B	B	B	B	B
5	4321335	10.722	rs155279	0.368		B				A		A			B	B		A
5	4325295	10.7221	rs155353	0.268					B	B		B			A	A		B
5	4327455	10.7221	rs155354	0.131	B	B	B	B	B	B		B		B				B
5	4336158	10.7248	rs16873316	0.013	B	B	B	B	B	B	B	B	B	B	B	B	B	B
5	4341114	10.7249	rs10069119	0.102	B	B	B	B	B	B	B	B	B	B	B	B	B	B
5	4346529	10.7258	rs155356	0.854	A		A	A	A	A	A	A	A	A	A	A	A	A
5	4355708	10.7268	rs17727202	0.915	A	A	A	A		A	A	A	A	A	A	A	A	A
5	4356792	10.727	rs261144	0.64	A		A	A		A	A	A	A	A	A	A	A	A
5	4362756	10.7288	rs13185799	0.097	B	B	B	B	B		B	B	B	B	B	B	B	B
5	4368462	10.7301	rs261124	0.248	B				B	B	B	B	B	B	B	B	B	B
5	4369887	10.7307	rs261125	0.522	A					A	A	A	A	A	A	A	A	A
5	4372548	10.7321	rs1354129	0.759	A	A	A	A		A	A	A	A	A	A	A	A	A
5	4375021	10.7334	rs9313075	0.128	B	B	B	B	B	B	B	B	B	B	B	B	B	B
5	4375160	10.7341	rs261126	0.296		B	B	B	B		B		B		B	B	B	
5	4375574	10.7429	rs261128	0.305	B				B	B	B	B	B	B	B	B	B	B
5	4375862	10.7508	rs13175033	0.597		A	A	A	A			A		A				A
5	4376022	10.7545	rs261129	0.58	B					B		B		B				
5	4376717	10.756	rs261130	0.854	A	A	A	A	A	A	A		A		A	A	A	A
5	4380545	10.7636	rs12187807	0.111		B	B	B	B		B	B	B	B	B	B	B	B
5	4380725	10.7648	rs4701855	0.743	B					B								
5	4381727	10.7652	rs10073943	1	A	A	A	A	A	A	A	A	A	A	A	A	A	A
5	4388200	10.7681	rs10052590	0	B	B	B	B	B	B	B	B	B	B	B	B	B	B
5	4389744	10.7687	rs11948367	1	A	A	A	A	A	A	A	A	A	A	A	A	A	A
5	4390768	10.7692	rs1080648	0.646	B					B								
5	4391653	10.7695	rs12716158	0.761	B					B								
5	4396901	10.7739	rs13168384	0.231		B	B	B	B		B	B	B	B	B	B	B	B
5	4397173	10.7807	rs9313076	0	B	B	B	B	B	B	B	B	B	B	B	B	B	B
5	4397964	10.8214	rs9313078	0.138	B	B			B	B	B	B	B	B	B	B	B	B
5	4399737	10.9523	rs17677148	0.332														
5	4405918	11.0716	rs7718259	0.929	A		A	A	A	A	A	A	A	A	A	A	A	A
5	4407484	11.0765	rs4702677	0.646			A		A	A				A	A	A	A	A
5	4409168	11.0843	rs4701864	0.155	B	B	B		B	B	B	B	B	B	B	B		B
5	4417155	11.0925	rs10061612	1	A	A	A	A	A	A	A	A	A	A	A	A	A	A
5	4417450	11.0926	rs12518914	0.801	A	A	A		A	A		A	A	A	A	A		A
5	4419552	11.0934	rs10044501	0.947	A	A	A	A	A	A	A	A	A	A	A	A	A	
5	4420716	11.0948	rs13186177	0.274														
5	4423292	11.0962	rs10078927	0.058	B	B	B	B	B	B	B	B	B	B	B	B	B	
5	4426699	11.0967	rs10076204	0.478														
5	4428580	11.097	rs11134052	0.221														
5	4429187	11.0972	rs10054113	0.947	A	A	A	A	A	A	A	A	A	A	A	A	A	
5	4436013	11.0991	rs4286636	0.947	A	A	A	A	A	A	A	A	A	A	A	A	A	
5	4438356	11.103	rs6894740	0.929	A	A	A	A	A	A	A	A	A	A	A	A	A	
5	4439260	11.1051	rs10064177	0.628	A	A	A	A	A		A	A	A		A	A	A	
5	4439651	11.1199	rs6861699	0.735	B	B			B			B						B
5	4439786	11.125	rs6887047	0.739	A				A	A		A	A	A				A
5	4440130	11.138	rs6887571	0.549		A		A	A	A	A		A	A			A	
5	4442390	11.2175	rs2043354	0.004	B	B	B	B	B	B	B	B	B	B	B	B	B	B
5	4455368	11.2594	rs4701877	0.73	A	A	A		A	A		A		A	A	A		A
5	4456379	11.2595	rs17276015	0	B	B	B	B	B	B	B	B	B	B	B	B	B	B
5	4456841	11.2595	rs1433271	0.996	A	A	A	A	A	A	A	A	A	A	A	A	A	A
5	4467380	11.2603	rs4701884	0	B	B	B	B	B	B	B	B	B	B	B	B	B	B
5	4474329	11.2689	rs9313082	0.305		A								A			A	
5	4474589	11.2696	rs1433265	0.71	A	A			A	A		A		A			A	A
5	4478547	11.2713	rs10066908	0.655										B			B	
5	4488309	11.2732	rs10462795	0.792	A	A	A		A	A		A		A	A	A	A	A
5	4491526	11.2745	rs10036724	1	A	A	A	A	A	A	A	A	A	A	A	A	A	A
5	4493944	11.2748	rs12516470	0.77	A	A			A	A		A		A			A	A
5	4510920	11.2768	rs16873498	0	B	B	B	B	B	B	B	B	B	B	B	B	B	B
5	4511087	11.2769	rs1450827	0.181	B	B	B		B	B		B		B	B	B	B	B
5	4512711	11.2826	rs921393	0	B	B	B	B	B	B	B	B	B	B	B	B	B	B
5	4514532	11.3006	rs16873509	0.162	B	B	B		B	B		B		B	B	B	B	B
5	4515447	11.3034	rs6881826	0.802	A	A			A	A		A		A			A	A
5	4515553	11.3037	rs921392	0.946	A	A		A	A	A	A	A	A	A			A	A
5	4516670	11.3057	rs11134055	0.681	A		A	A		A	A	A	A		A	A		
5	4517509	11.3072	rs13187596	0.679	A		A	A		A	A	A	A		A	A		
5	4520220	11.3399	rs12517115	0.177	B	B	B		B	B		B		B	B	B	B	B
5	4520856	11.3481	rs1450822	0.509				B			B		B	B			B	B
5	4532227	11.354	rs17690482	0.212	B	B	B		B	B		B		B	B	B	B	B
5	4534191	11.3553	rs6555299	0.261	B	B	B	B	B	B	B	B	B		B	B		B
5	4542749	11.3743	rs3943533	0.735	A	A	A		A	A		A		A	A	A	A	
5	4547644	11.3882	rs10070988	0	B	B	B	B	B	B	B	B	B	B	B	B	B	B
5	4547704	11.3882	rs10064215	0.294	B	B			B	B		B		B			B	
5	4565002	11.3949	rs10474781	0	B	B	B	B	B	B	B	B	B	B	B	B	B	B
5	4568974	11.3956	rs12515499	0.712	A	A			A	A		A		A			A	
5	4574945	11.4474	rs1030490	0.954	A		A	A	A	A	A	A	A	A	A	A	A	A
5	4576516	11.4699	rs7700360	0.208	B	B		B	B			B					B	
5	4577399	11.4763	rs2052887	0.308	B			B	B			B					B	
5	4577842	11.477	rs12657445	0.377	B	B		B	B			B					B	
5	4578875	11.4786	rs16873639	0.761	A	A	A	A	A			A			A	A	A	
5	4593655	11.5929	rs10063610	0	B	B	B	B	B	B	B	B	B	B	B	B	B	B
5	4599400	11.5951	rs293067	0.283				B	B		B		B		B	B	B	
5	4600961	11.5956	rs7730075	0.796				A	A		A	A	A		A	A	A	
5	4601882	11.596	rs7710837	0.005	B	B	B	B	B	B	B	B	B	B	B	B	B	B
5	4605931	11.5976	rs6894400	1	A	A	A	A	A	A	A	A	A	A	A	A	A	A
5	4610206	11.5997	rs16873676	0.008	B	B	B	B	B	B	B	B	B	B	B	B	B	B
5	4629210	11.6294	rs6887582	0.293		A			A	A								
5	4632694	11.7446	rs293102	0.35	B	B	B	B	B	B		B	B	B	B	B	B	B
5	4633275	11.7711	rs293105	0.487			B		B			B	B					
5	4633409	11.7732	rs10512742	0.081	B	B	B	B	B	B	B	B	B	B	B	B	B	B
5	4633984	11.778	rs10512743	0.062	B	B	B	B	B	B	B	B	B	B	B	B	B	B
5	4634162	11.7788	rs293106	0.389			A		A			A	A					
5	4638885	11.7855	rs7713961	0.867	A	A	A	A	A	A		A	A	A	A	A	A	A
5	4641160	11.7898	rs293119	0.4			B		B	B	B	B	B					
5	4644270	11.7957	rs293123	0.593					B	B		B	B					
5	4644642	11.7968	rs11134061	0.217					A			A	A					
5	4645455	11.8001	rs2077369	0.093	B	B	B	B	B	B	B	B	B	B	B	B	B	B
5	4647436	11.8107	rs7713507	0.156	B	B	B	B	B	B	B	B	B	B	B	B	B	B
5	4647464	11.8108	rs6555302	0.754	A	A	A	A	A	A	A	A	A	A	A	A	A	A
5	4647493	11.8109	rs7713659	0.296	B	B	B	B	B	B	B	B	B	B	B	B	B	B
5	4648002	11.8132	rs418752	0.845	A	A	A	A	A		A	A	A	A	A	A	A	A
5	4648027	11.8133	rs1428981	0.58			B		B			B	B					
5	4651632	11.8176	rs16873742	0.951	A	A	A	A	A	A	A	A	A	A		A	A	A
5	4651742	11.8177	rs450409	0.923	A	A	A	A	A	A	A	A	A	A	A	A	A	A
5	4654214	11.8268	rs1366642	0.518			A		A			A	A	A				
5	4654629	11.829	rs17692869	0.562			A		A			A	A	A				

Chromosome	Physical position (hg19)	Genetic map	rs number	Allele A frequency	Family 2 (GC15626)		Family 3 (GC15119)				Family 4 (GC13840)			Family 5 (GC19075)			Family 6 (GC15475)	Family 7 (GC11709)
					IV:5	IV:6	IV:III	IV:II	III:1	V:1	II:1	I:1	II:2	III:4	II:3	II:2	I:1	II:1
5	4657375	11.8314	rs1366643	0.92	A	A		A	A	A	A	A	A	A	A	A	A	A
5	4657945	11.8319	rs1346575	0.252	B	B		B	B	B	B	B	B		B	B	B	B
5	4659940	11.8345	rs3928432	0.73	A	A		A	A		A	A	A		A	A	A	A
5	4660902	11.8359	rs2115525	0.513			A		A	A		A	A	A				
5	4662323	11.8373	rs7447371	0.987	A	A	A	A	A	A	A	A	A	A	A	A	A	A
5	4663347	11.8414	rs4702895	0.08	B	B	B	B	B	B	B	B	B		B	B	B	B
5	4664704	11.8426	rs2162995	0.125	B	B	B	B	B	B	B	B	B		B	B	B	B
5	4664870	11.8427	rs11744458	0.097	B	B		B	B		B	B	B	B	B	B	B	B
5	4665354	11.8432	rs16873806	0.031	B	B	B	B	B	B	B	B	B	B	B	B	B	B
5	4665670	11.8437	rs4370242	0.527			A		A	A			A	A				
5	4665724	11.8438	rs2895497	0.424					A			A	A	A				
5	4668289	11.8603	rs4407602	0.792	A			A	A		A	A	A	A	A	A	A	A
5	4668593	11.8618	rs7731649	0.235					A			A	A					
5	4670864	11.8647	rs7724465	0.058	B			B	B	B	B	B	B	B	B	B	B	B
5	4671383	11.8653	rs6555306	0.111	B			B	B	B	B	B	B	B	B	B	B	B
5	4673835	11.8872	rs7720816	0.429	B	B	B	B		B	B	B	B					
5	4674868	11.9199	rs4412086	0.425	A	A	A	A				A	A					
5	4675355	11.9355	rs7716432	0.549	A	A	A	A		A	A	A	A					
5	4676429	11.9564	rs4568336	0.987	A	A	A	A	A		A	A	A	A	A	A	A	A
5	4677722	11.9819	rs13359697	0.172	B		B	B	B	B		B	B	B	B	B		
5	4678192	11.983	rs7713482	0.607					A			A	A	A	A	A		A
5	4678221	11.9831	rs10063789	0.836	A		A	A	A	A		A	A	A	A	A		A
5	4678287	11.9831	rs7718402	0.934	A	A	A	A	A	A	A	A	A	A	A	A	A	A
5	4679559	11.9836	rs4235620	0.73		A			A		A	A	A	A	A	A	A	A
5	4680691	11.9839	rs4563594	0.119	B	B	B	B	B	B	B	B	B	B	B	B	B	B
5	4692090	11.9878	rs11134066	0	B	B	B	B	B	B	B	B	B	B	B	B	B	B
5	4695085	11.9904	rs6885274	0.938	A	A	A		A		A	A	A	A	A	A	A	A
5	4700587	11.9962	rs4702913	0.164		B			B		B	B	B	B	B	B	B	B
5	4705982	11.9983	rs12716162	0.854	A	A	A	A	A	A		A	A	A	A	A	A	A
5	4706519	11.9985	rs4532332	0.805		A			A		A	A	A	A	A	A	A	A
5	4717060	11.9999	rs2047728	0.585		A			A			A	A	A	A	A	A	A
5	4723324	12.002	rs6890255	0.954	A	A	A	A	A	A	A	A	A	A	A	A	A	A
5	4724332	12.0034	rs7711434	0.832		A	A	A	A	A		A	A	A	A	A	A	A
5	4725405	12.0044	rs10035433	0.889	A	A	A	A	A	A	A	A	A	A	A	A	A	A
5	4727057	12.0049	rs17750180	0.934	A	A	A	A	A	A		A	A	A	A	A	A	A
5	4730319	12.0088	rs6895818	0.915	A	A	A	A	A	A	A	A	A	A	A	A	A	A
5	4734171	12.0224	rs2134180	0.356		B	B		B	B		B	B	B	B	B	B	B
5	4738222	12.023	rs1393120	0.1	B	B	B	B	B	B	B	B	B	B	B	B	B	B
5	4755398	12.0281	rs7716642	0.827	B			B			B							
5	4761704	12.0313	rs1501760	0.699	B			B			B							
5	4776191	12.0387	rs2088900	0.341		B	B			B			B	B	B	B	B	B
5	4776500	12.0407	rs4235550	0.893				B										
5	4777480	12.0466	rs10069581	0.093	B	B	B	B	B	B	B	B	B	B	B	B	B	B
5	4780629	12.0758	rs16874044	0.15	B	B	B	B	B	B	B	B	B	B	B	B	B	B
5	4781079	12.0786	rs1501754	1	A	A	A	A	A	A	A	A	A	A	A	A	A	A
5	4784657	12.0795	rs1173229	0.239		B	B		B	B	B	B	B	B	B	B	B	B
5	4785389	12.0797	rs1173228	0.717		A	A		A	A	A	A	A	A	A	A	A	A
5	4785594	12.0797	rs1173227	0.717		A	A		A	A	A	A	A	A	A	A	A	A
5	4788892	12.0809	rs1173218	0.279		B	B		B	B	B	B	B	B	B	B	B	B
5	4795354	12.0894	rs1452040	0.754		A	A		A	A	A	A	A	A	A	A	A	A
5	4801119	12.1016	rs10051649	0.283		B	B		B	B	B	B	B	B	B	B	B	B
5	4801996	12.1209	rs7728112	0.947	A	A	A	A	A	A	A	A	A	A	A	A	A	A
5	4803549	12.1286	rs1173179	0.597		A					A		A					A
5	4814945	12.1543	rs1173192	0.173	B	B	B	B	B	B	B	B	B	B	B	B	B	B
5	4819918	12.1554	rs1173201	0.112	B	B	B	B	B	B	B	B	B	B	B	B	B	B
5	4821758	12.1554	rs10063051	0.004	B	B	B	B	B	B	B	B	B	B	B	B	B	B
5	4824652	12.1556	rs10036366	0	B	B	B	B	B	B	B	B	B	B	B	B	B	B
5	4825401	12.1556	rs1173213	0.814	A	A	A	A	A	A	A	A	A	A	A	A	A	A
5	4827086	12.1559	rs10073827	0	B	B	B	B	B	B	B	B	B	B	B	B	B	B
5	4828015	12.156	rs11134077	0.05	B	B	B	B	B	B	B	B	B	B	B	B	B	B
5	4831601	12.1563	rs816475	0.889	A	A	A	A	A	A	A	A	A	A	A	A	A	A
5	4831687	12.1563	rs2123104	0.94	A	A	A	A	A	A	A	A	A	A	A	A	A	A
5	4835205	12.1602	rs816480	0.124	B	B	B	B	B	B	B	B	B	B	B	B	B	B
5	4839617	12.1611	rs10512749	0.062	B	B	B	B	B	B	B	B	B	B	B	B	B	B
5	4840284	12.1612	rs2652227	0.071	B	B	B	B	B	B	B	B	B	B	B	B	B	B
5	4844322	12.1617	rs2652221	0.969	A	A	A	A	A	A	A	A	A	A	A	A	A	A
5	4853391	12.1627	rs860987	0.876	A	A	A	A	A	A	A	A	A	A	A	A	A	A
5	4861432	12.1631	rs2600642	0.005	B	B	B	B	B	B	B	B	B	B	B	B	B	B
5	4867913	12.1653	rs707645	0.869	A	A	A	A	A	A		A	A	A	A	A	A	A
5	4872310	12.1678	rs816467	0.062	B	B	B	B	B	B		B	B	B	B	B	B	B
5	4872511	12.1679	rs816468	0.841	A	A	A	A	A	A		A	A	A	A	A	A	A
5	4875420	12.1702	rs16874171	0.048	B	B	B	B	B	B	B	B	B	B	B	B	B	B
5	4884392	12.1738	rs7728954	0.938	A	A	A	A	A	A	A	A	A	A	A	A	A	A
5	4885068	12.1745	rs629342	0.478		A				A	A							
5	4885562	12.1753	rs17752880	0.442		A				A			A					A
5	4887171	12.1769	rs10051514	0.403		B				B	B		B					B
5	4888934	12.1797	rs11857958	0.554														
5	4900062	12.1975	rs590199	0.08	B	B	B	B	B	B		B	B	B	B	B	B	B
5	4901816	12.2051	rs26875	0.836	A	A	A	A	A	A		A	A	A	A	A	A	A
5	4905892	12.2402	rs26880	0.535	B	B	B	B	B			B		B	B	B	B	
5	4907084	12.2483	rs6894984	0.06	B	B	B	B	B	B		B	B	B	B	B	B	B
5	4907778	12.252	rs1392411	0.562	A	A	A	A	A		A	A		A	A	A	A	
5	4912310	12.261	rs610722	0.372	A		A	A	A			A		A			A	
5	4912405	12.2611	rs9885575	0	B	B	B	B	B	B	B	B	B	B	B	B	B	B
5	4913228	12.2628	rs6879578	0.991	A	A	A	A	A	A	A	A	A	A	A	A	A	A
5	4914886	12.2661	rs6555321	0.487	A	A	A	A	A		A	A		A			A	
5	4917365	12.2709	rs484696	0.314	B		B		B	B		B	B		B	B	B	B
5	4924195	12.2734	rs10475277	0	B	B	B	B	B	B	B	B	B	B	B	B	B	B
5	4928318	12.2752	rs501031	0.681	A		A		A	A		A	A		A	A	A	A
5	4928520	12.2754	rs502854	0.308	B		B		B	B		B	B		B	B	B	B
5	4930407	12.2824	rs30452	0.664	A		A		A	A	A	A	A		A	A	A	A
5	4936235	12.2956	rs654685	0.708					A	A		A	A					A
5	4937880	12.2981	rs12521477	0.738					A	A		A	A					A
5	4938130	12.2987	rs609096	0.814		A	A		A	A	A	A	A				A	A
5	4938756	12.3013	rs596041	0.032	B	B	B	B	B	B	B	B	B	B	B	B	B	B
5	4938915	12.3021	rs6868240	0.886	A			A	A	A		A	A	A	A	A		A
5	4941242	12.3567	rs11134086	0.746		A	A	A		A	A		A				A	
5	4945124	12.3657	rs30448	0.996	A	A	A	A	A	A	A	A	A	A	A	A	A	A
5	4946412	12.3664	rs30445	0.73		A	A	A		A	A		A				A	A
5	4948424	12.3685	rs643466	0.27		B	B	B		B	B		B				B	B
5	4948859	12.3866	rs466277	0.881		A	A	A	A	A	A		A		A	A	A	A
5	4950651	12.4005	rs458279	0.69			A	A	A	A			A		A	A	A	A
5	4951614	12.4019	rs26847	0.69			A	A	A	A			A		A	A	A	A
5	4953073	12.4051	rs7736239	0	B	B	B	B	B	B	B	B	B	B	B	B	B	B
5	4955950	12.4064	rs10070917	0.75			A	A	A	A			A		A	A	A	A
5	4957205	12.4072	rs891001	1	A	A	A	A	A	A	A	A	A	A	A	A	A	A
5	4959279	12.4211	rs1366431	0.372			B	B	B	B					B	B	B	B
5	4966602	12.4509	rs1452028	1	A	A	A	A	A	A	A	A	A	A	A	A	A	A
5	4968029	12.4512	rs1452027	0.35		B	B	B		B	B						B	B
5	4971022	12.4515	rs16874489	1	A	A	A	A	A	A	A	A	A	A	A	A	A	A
5	4977446	12.4523	rs42202	0.106	B	B	B	B	B	B	B	B	B	B	B	B	B	B
5	4978270	12.4531	rs42401	0.688		A	A	A		A	A						A	A
5	4987376	12.4577	rs30463	0.65		A	A	A		A	A						A	A
5	4992930	12.459	rs30449	0.642		A	A	A		A	A						A	A
5	5003664	12.4901	rs7734957	1	A	A	A	A	A	A	A	A	A	A	A	A	A	A
5	5009457	12.5053	rs30485	0.916	A	A	A	A	A	A	A	A		A	A	A	A	A
5	5023441	12.5088	rs458084	0.487					A				A					A
5	5023538	12.5088	rs16874586	0.031	B	B	B	B	B	B	B	B	B	B	B	B	B	B
5	5023977	12.5091	rs467075	0.942	A	A	A	A	A	A	A	A	A	A	A	A	A	A
5	5024621	12.5095	rs465871	0.456					A				A					A

Chromosome	Physical position (hg19)	Genetic map	rs number	Allele A frequency	Family 2 (GC15626)		Family 3 (GC15119)				Family 4 (GC13840)			Family 5 (GC19075)			Family 6 (GC15475)	Family 7 (GC11709)
					IV:5	IV:6	IV:III	IV:II	III:1	V:1	II:1	I:1	II:2	III:4	II:3	II:2	I:1	II:1
5	5032533	12.5136	rs6878673	0.426					A				A					A
5	5037512	12.5344	rs16874610	0.031	B	B	B	B	B	B	B	B		B	B	B	B	B
5	5038540	12.535	rs446464	0.075	B	B	B	B	B	B	B	B		B			B	B
5	5051464	12.5449	rs272194	0.692	A	A	A	A	A	A	A	A		A			A	A
5	5052953	12.5455	rs16874638	0.031														
5	5052998	12.5455	rs272195	0.31	B	B	B	B	B	B	B	B		B			B	B
5	5062490	12.5524	rs272162	0.053	B	B	B	B	B	B	B	B	B	B			B	B
5	5063242	12.5527	rs156251	0.894	B									B	B	B		
5	5072083	12.5751	rs270642	0.977	A	A	A	A	A	A	A	A	A				A	A
5	5074825	12.5776	rs270636	0.381	B	B	B						B	B	B	B	B	B
5	5078313	12.5802	rs3733787	0.438		A	A						A				A	
5	5080435	12.5816	rs6866197	1	A	A	A	A	A	A	A	A	A	A	A	A	A	A
5	5084233	12.5845	rs899556	0.602	A	A	A						A	A	A	A	A	A
5	5089229	12.5852	rs1382896	0.592	A	A	A						A	A	A	A	A	A
5	5093172	12.5868	rs2619799	0.602	A	A	A						A	A	A	A	A	A
5	5093643	12.5869	rs1160800	0.602	A	A	A						A	A	A	A	A	A
5	5098840	12.5889	rs814797	0.379	B	B	B			B			B				B	B
5	5106180	12.5909	rs16874709	0.858		A	A	A	A	A	A	A	A	A	A	A	A	
5	5110692	12.5955	rs814790	0.367						A			A				A	
5	5116544	12.7194	rs2115010	0.177	B	B	B	B	B	B	B	B	B	B	B	B	B	B
5	5120745	12.7211	rs814783	0.65			A	A		A		A	A	A				
5	5120776	12.7211	rs861512	0.465			A	A		A			A	A				
5	5121337	12.7213	rs1834925	0.819	A	A	A	A	A	A	A	A	A	A	A	A	A	A
5	5123214	12.7221	rs9313102	0.19	B	B	B	B	B	B	B		B	B	B	B	B	B
5	5124149	12.7227	rs270188	0.845	A		A	A		A	A	A	A	A				A
5	5124263	12.7229	rs270187	0.358			B	B		B			B	B				
5	5124549	12.7237	rs16874791	0.805		A	A	A	A	A		A	A	A	A	A	A	
5	5125959	12.7287	rs10475286	0.819	A	A	A	A	A	A	A	A	A	A	A	A	A	A
5	5126400	12.7313	rs10042501	0.181	B	B	B	B	B	B	B	B	B	B	B	B	B	B
5	5128141	12.7434	rs814776	0.035														
5	5129879	12.7678	rs12516403	0.805		A	A	A	A	A		A	A	A	A	A	A	
5	5133444	12.8448	rs751905	0.664								B		B				
5	5136207	12.9044	rs7720806	0.699			A		A			A	A	A			A	
5	5140970	13.0286	rs270206	0.712		A			A		A		A	A				A
5	5141905	13.0529	rs270198	0.427	B	B						B					B	B
5	5142460	13.054	rs2291112	0.376	B	B			B			B					B	B
5	5142768	13.0542	rs270193	0.611	A	A			A			A					A	A
5	5145406	13.056	rs1863970	0.273	B	B		B	B	B		B			B	B	B	B
5	5146395	13.0574	rs1863968	0.381	B	B			B			B					B	B
5	5147125	13.0587	rs10512769	0.836	A	A	A	A	A	A	A	A	A	A	A	A	A	A
5	5147376	13.0592	rs2255273	0.894	A	A	A		A		A	A	A	A			A	A
5	5149621	13.0607	rs2560412	0.212	B	B		B	B	B		B			B	B	B	B
5	5152447	13.0612	rs11134095	0.156	B	B	B		B		B	B	B	B			B	B
5	5153648	13.062	rs978280	0.549	B	B			B			B					B	B
5	5153911	13.0623	rs16874878	0.186	B	B	B		B		B	B	B	B			B	B
5	5154215	13.0626	rs922542	0.637	A	A		A	A	A		A			A	A	A	A
5	5154458	13.0634	rs6555331	0.544	B	B			B			B					B	B
5	5155357	13.0666	rs814775	0.681	A	A	A	A	A	A		A			A	A	A	A
5	5156655	13.0686	rs6899080	0.673	B	B			B			B					B	
5	5159316	13.0708	rs7720897	0.217	B	B		B	B	B		B			B	B	B	B
5	5160481	13.0717	rs10067690	0.155	B	B	B	B	B	B	B	B	B	B	B	B	B	B
5	5162228	13.073	rs992542	0.73	A	A		A	A	A		A			A	A	A	A
5	5164175	13.0745	rs7705998	0.941	A	A	A	A	A	A	A	A	A	A	A	A	A	A
5	5165415	13.076	rs270178	0.513	B	B			B			B					B	
5	5165870	13.0835	rs874428	0.161		B	B	B		B	B		B	B	B	B		B
5	5165952	13.0849	rs1079295	0.354	B	B		B	B	B		B			B	B	B	B
5	5167587	13.0926	rs997427	0.45	B	B		B	B	B		B			B	B	B	B
5	5167648	13.0927	rs997428	0.876	A	A	A	A	A	A	A	A	A	A	A	A	A	A
5	5176672	13.1156	rs4701693	0.129	B	B	B	B	B	B		B		B	B	B	B	B
5	5178624	13.117	rs4702234	0.615		A	A	A	A	A				A	A	A		A
5	5181634	13.1192	rs13177816	0.366		A	A							A				
5	5181829	13.1196	rs13176907	0.365		A	A							A				
5	5181928	13.1203	rs7724547	0.18	B	B	B				B	B	B	B			B	
5	5183700	13.1522	rs1808529	0.792	A	A		A	A	A	A	A		A	A	A	A	A
5	5183783	13.1538	rs10037656	0.907	A	A	A	A	A	A	A	A		A	A	A	A	A
5	5184252	13.1565	rs12518196	0.245		B	B	B	B	B	B		B		B	B		B
5	5187371	13.1636	rs4702235	0.226	B	B	B	B	B	B	B	B	B	B	B	B	B	B
5	5191047	13.1644	rs11743270	0.96	A	A	A	A	A	A	A	A	A	A	A	A	A	A
5	5191117	13.1645	rs12517136	0.906	A	A	A	A	A	A	A	A	A	A	A	A	A	A
5	5192434	13.1647	rs12520783	0.093	B	B	B	B	B	B	B	B	B	B	B	B	B	B
5	5192861	13.1648	rs871122	0.115	B	B	B	B	B	B	B	B		B	B	B	B	B
5	5195069	13.1652	rs10039354	0.221	B	B	B					B	B	B			B	
5	5195384	13.1658	rs4701694	0.208	B	B	B	B	B	B	B	B	B	B	B	B	B	B
5	5195721	13.1666	rs16874994	0.907	A	A	A	A	A	A	A	A	A	A	A	A	A	A
5	5196007	13.1671	rs2081852	0.867	A	A	A	A	A	A	A	A	A	A	A	A	A	A
5	5197553	13.1693	rs3943879	0.419	A	A	A					A		A			A	
5	5198567	13.1697	rs16875033	0.093	B	B	B	B	B	B	B	B	B	B	B	B	B	B
5	5199350	13.1701	rs1346460	0.167	B	B	B	B	B	B	B	B	B	B	B	B	B	B
5	5199787	13.1703	rs1428027	0.106	B	B	B	B	B	B	B	B	B	B	B	B	B	B
5	5200281	13.1709	rs6555335	0.804	A	A	A	A	A	A	A	A	A	A	A	A	A	A
5	5200490	13.1717	rs17762877	0.576	B	B	B					B		B			B	
5	5202232	13.1735	rs4702241	0.155	B	B	B	B	B	B	B	B		B	B	B	B	B
5	5202753	13.1757	rs979708	0.783	A	A	A	A	A	A	A	A	A	A	A	A	A	A
5	5203046	13.1768	rs979710	0.876	A	A	A	A	A	A	A	A	A	A	A	A	A	A
5	5203600	13.1775	rs1428024	0.907	A	A	A	A	A	A	A	A	A	A	A	A	A	A
5	5204149	13.1783	rs1366165	0.296	B	B	B					B	B	B			B	
5	5208254	13.2474	rs6888758	0.584	A		A		A	A	A	A	A	A			A	A
5	5209210	13.3266	rs16875054	0.027	B	B	B	B	B	B	B	B	B	B	B	B	B	B
5	5209872	13.3627	rs2019251	0.721	A	A			A		A	A	A	A	A	A	A	A
5	5210050	13.3629	rs1560063	0.735	A	A	A	A	A	A		A		A			A	A
5	5210315	13.3631	rs7704912	0.339			B	B		B	A				B	B	B	
5	5214077	13.3652	rs1019748	0.35			B	B		B		A			B	B	B	
5	5221300	13.3711	rs1560062	0.863							A	A	A					
5	5222336	13.3727	rs4702248	0.54			B	B		B		A	A					
5	5224299	13.3742	rs16875084	0.19	B	B	B	B	B	B	B	B		B			B	
5	5225032	13.3743	rs2964444	0.327	B	B			B			B	B	B	B	B	B	B
5	5226211	13.3744	rs2913615	0.655			A	A		A		B			A	A		A
5	5227291	13.3745	rs12652633	0.934	A	A	A	A	A	A		A	A	A	A	A	A	A
5	5227314	13.3745	rs16875090	0.863							A	A	A					
5	5232398	13.3775	rs2913624	0.673	A	A			A			A	A	A	A	A	A	A
5	5234261	13.3834	rs10067097	0.562			B	B		B	A	A	A					
5	5236356	13.3875	rs2913633	0.491	A	A			A		A	A		A			A	
5	5237807	13.3891	rs1991459	0.628			A	A		A	B	B			A	A		A
